# HOXA9 promotes MYC-mediated leukemogenesis by maintaining gene expression for multiple anti-apoptotic pathways

**DOI:** 10.7554/eLife.64148

**Published:** 2021-07-26

**Authors:** Ryo Miyamoto, Akinori Kanai, Hiroshi Okuda, Yosuke Komata, Satoshi Takahashi, Hirotaka Matsui, Toshiya Inaba, Akihiko Yokoyama

**Affiliations:** 1 Tsuruoka Metabolomics Laboratory, National Cancer CenterTsuruokaJapan; 2 Department of Molecular Oncology and Leukemia Program Project, Research Institute for Radiation Biology and Medicine, Hiroshima UniversityHiroshimaJapan; 3 Department of Hematology and Oncology, Kyoto University Graduate School of MedicineKyotoJapan; 4 Department of Molecular Laboratory Medicine, Graduate School of Medical Sciences, Kumamoto UniversityKumamotoJapan; 5 Division of Hematological Malignancy, National Cancer Center Research InstituteTokyoJapan; Fox Chase Cancer CenterUnited States; Fox Chase Cancer CenterUnited States

**Keywords:** HOXA9, MYC, leukemia, apoptosis, transcription, Mouse

## Abstract

HOXA9 is often highly expressed in leukemias. However, its precise roles in leukemogenesis remain elusive. Here, we show that HOXA9 maintains gene expression for multiple anti-apoptotic pathways to promote leukemogenesis. In MLL fusion-mediated leukemia, MLL fusion directly activates the expression of MYC and HOXA9. Combined expression of MYC and HOXA9 induced leukemia, whereas single gene transduction of either did not, indicating a synergy between MYC and HOXA9. HOXA9 sustained expression of the genes implicated in the hematopoietic precursor identity when expressed in hematopoietic precursors, but did not reactivate it once silenced. Among the HOXA9 target genes, *BCL2* and *SOX4* synergistically induced leukemia with *MYC*. Not only BCL2, but also SOX4 suppressed apoptosis, indicating that multiple anti-apoptotic pathways underlie cooperative leukemogenesis by HOXA9 and MYC. These results demonstrate that HOXA9 is a crucial transcriptional maintenance factor that promotes MYC-mediated leukemogenesis, potentially explaining why HOXA9 is highly expressed in many leukemias.

## Introduction

Mutations of transcriptional regulators often cause aberrant gene regulation of hematopoietic cells, which leads to leukemia. Structural alterations of the mixed lineage leukemia gene (*KMT2A* also known as *MLL*) by chromosomal translocations cause malignant leukemia that often associates with poor prognosis despite the current intensive treatment regimens (Tamai and Inokuchi, 2010). *KMT2A* encodes a transcriptional regulator termed MLL that maintains segment-specific expression of homeobox (*HOX*) genes during embryogenesis (Yu et al., 1998), which determines the positional identity within the body (Luo et al., 2019; Deschamps and van Nes, 2005; Wang et al., 2009). During hematopoiesis, MLL also maintains the expression of posterior *HOXA* genes and *MEIS1* (another homeobox gene), which promote the expansion of hematopoietic stem cells and immature progenitors (Jude et al., 2007; Krivtsov et al., 2006; McMahon et al., 2007; Thorsteinsdottir et al., 2002; Yagi et al., 1998). The oncogenic MLL fusion protein constitutively activates its target genes by constitutively recruiting transcription initiation/elongation factors thereto (Yokoyama et al., 2010; Lin et al., 2010; Okuda et al., 2015). Consequently, *HOXA9* and *MEIS1* are highly transcribed in MLL fusion-mediated leukemia (Krivtsov et al., 2006). Forced expression of HOXA9 (but not MEIS1) immortalize hematopoietic progenitor cells (HPCs) ex vivo (Schnabel et al., 2000; Kroon et al., 1998). Co-expression of HOXA9 with MEIS1 causes leukemia in mice which recapitulates MLL fusion-mediated leukemia (Kroon et al., 1998). Moreover, overexpression of HOXA9 is observed in many non-MLL fusion-mediated leukemias such as those with *NPM1* mutation and *NUP98* fusion and is associated with poor prognosis (Collins and Hess, 2016). These findings highlight HOXA9 as a major contributing factor in leukemogenesis. Nevertheless, the mechanism by which HOXA9 promotes oncogenesis remains elusive.

HOXA9 is considered to function as a transcription factor, which retains a sequence-specific DNA binding ability. HOX proteins have an evolutionally conserved homeodomain which possesses strong sequence preferences (Berger et al., 2008). HOXA9 associates with other homeodomain proteins such as PBX and MEIS family proteins (Schnabel et al., 2000; Shen et al., 1999). HOXA9 and those HOXA9 cofactors form a stable complex on a DNA fragment harboring consensus sequences for each homeodomain protein (Shen et al., 1999; Chang et al., 1996), suggesting that they form a complex of different combinations in a locus-specific manner depending on the availability of the binding sites. Recently, it has been reported that HOXA9 specifically associates with enhancer apparatuses (e.g. MLL3/4) to regulate gene expression (Sun et al., 2018; Huang et al., 2012; Zhong et al., 2018). However, the mechanisms by which HOXA9 activates gene expression remain largely unclear.

In this study, we reveal the oncogenic roles for HOXA9 and its target gene products in leukemogenesis and its unique mode of function as a transcriptional maintenance factor that preserves an identity of a hematopoietic precursor.

## Results

### MLL fusion proteins and HOXA9 sustain MYC expression against differentiation-induced transcriptional suppression

**Figure 1.**
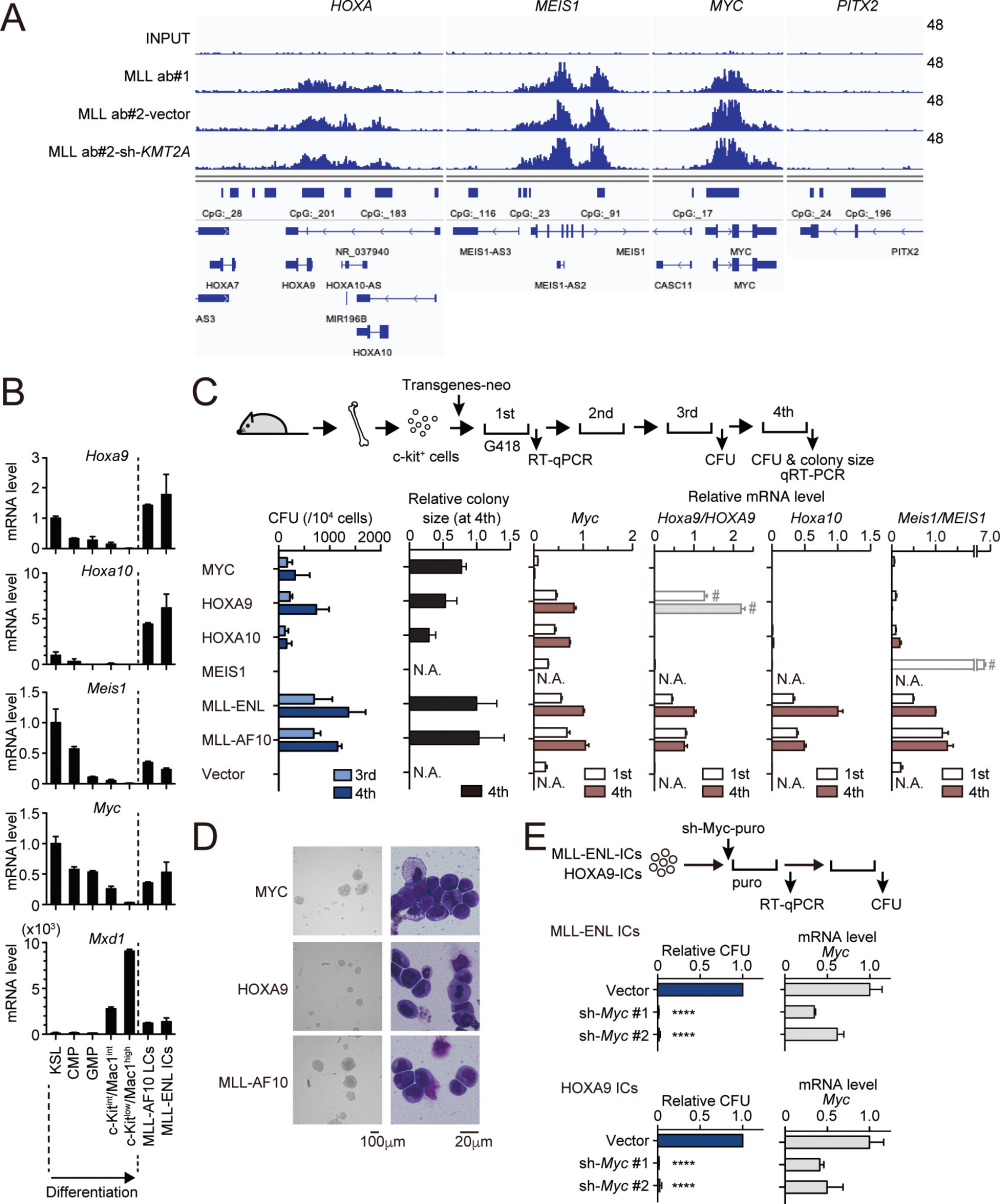
MLL fusion proteins and HOXA9 sustain MYC expression against differentiation-induced transcriptional suppression. (**A**) Genomic localization of MLL-ENL in HB1119 cells. ChIP signals at the loci of posterior *HOXA* genes, *MEIS1*, *MYC*, and *PITX2* (negative control) are shown using the Integrative Genomics Viewer (The Broad Institute). HB1119 cells were transduced with shRNA specific for wild-type (wt) MLL but not MLL-ENL (sh- *KMT2A*) to deplete wt MLL, as shown in Figure 1—figure supplement 1B. Ab: antibody. (**B**) Expression of MLL-target genes and *Mxd1* (a differentiation marker) during myeloid differentiation. Bone marrow cells at various differentiation stages were obtained by FACS sorting and analyzed by qRT-PCR. Expression levels relative to KSL are shown (Mean with SD, n = 3, PCR replicates). MLL-AF10-LCs and MLL-ENL-ICs were included in the analysis for comparison. KSL: c-Kit^+^, Sca1^+^, and Lineage^–^; CMP: common myeloid progenitor; GMP: granulocyte macrophage progenitor; int: intermediate. (**C**) Transforming potential of MLL target genes. Clonogenic potential of the indicated constructs was analyzed by myeloid progenitor transformation assays. Colony forming unit per 10^4^ cells (CFU) (Mean with SD, n = 3, biological replicates), relative colony size (Mean with SD, n ≥ 100), and relative mRNA levels of indicated genes (Mean with SD, n = 3, PCR replicates) were measured at the indicated time points. #: Both endogenous murine transcripts and exogenous human transcripts were detected by the qPCR primer set used and the columns were shown with faded color. N.A.: not assessed. (**D**) Morphologies of the colonies and transformed cells. Representative images of bright field (left) and May-Grunwald-Giemsa staining (right) are shown with scale bars. (**E**) Effects of *Myc* knockdown on MLL-ENL- and HOXA9-ICs. Relative CFU (Mean with SD, n = 3, biological replicates) and mRNA level of *Myc* (Mean with SD, n = 3 PCR replicates) are shown. Statistical analysis was performed using ordinary one-way ANOVA with the vector control. ****p < 0.0001.

To identify the direct target genes of MLL fusion proteins, we first examined the genome-wide localization pattern of MLL fusion proteins by chromatin immunoprecipitation (ChIP) followed by deep sequencing (ChIP-seq), using HB1119 cells, a cell line expressing the MLL-ENL fusion protein. We observed MLL ChIP signals on the *MYC*, *HOXA9*, *HOXA10*, and *MEIS1* loci (Figure 1A; Okuda et al., 2017), which were further confirmed by ChIP-quantitative polymerase chain reaction (qPCR) analysis (Figure 1—figure supplement 1A). These ChIP signals can be mostly attributed to MLL-ENL as the knockdown of wild-type MLL did not affect the MLL ChIP signals (Figure 1A and Figure 1—figure supplement 1B,C; Okuda et al., 2017). The distribution of MLL-ENL was enriched at transcription start sites in a genome-wide manner, which is similar to that of wild-type MLL observed in non-MLL fusion cell lines including HEK293T and REH (Okuda et al., 2017; Miyamoto et al., 2020).

**Figure 1—figure supplement 1.**
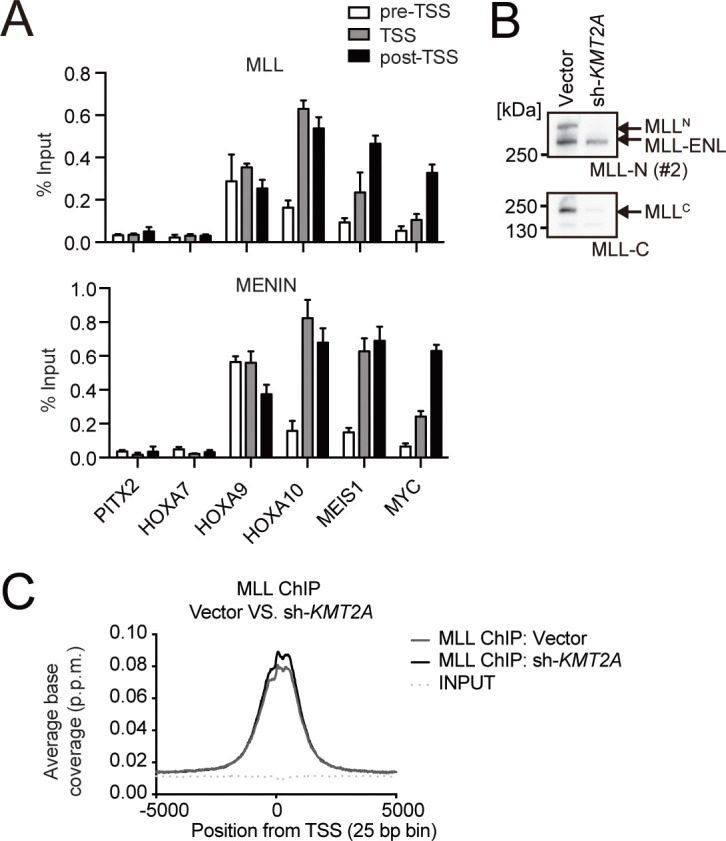
Association of the MLL-ENL complex on target promoters. (**A**) Genomic localization of the MLL-ENL complex in HB1119 cells. ChIP-qPCR was performed using anti-MLL and MENIN antibodies. Precipitated DNA was subjected to qPCR using specific probes for the pre-TSS (−1.0 to −0.5 kb from the TSS), TSS (0 to 0.5 kb from the TSS), and post-TSS (1.0 to 1.5 kb from the TSS) regions of the indicated genes. ChIP signals were expressed as the percent input (Mean with SD, n = 3, PCR replicates). (**B**) Depletion of wildtype (wt) MLL in HB1119 cells. HB1119 cells were transduced with shRNA specific for wt MLL, but not MLL-ENL (sh-*KMT2A*). Protein expression of MLL-ENL and wt MLL was analyzed by western blotting using specific antibodies against the N-terminal epitope of wt MLL, which is retained by both the MLL^N^ fragment of wt MLL and MLL-ENL, and the C-terminal epitope, which is retained only by the MLL^C^ fragment of wt MLL (Yokoyama et al., 2002). (**C**) Distribution patterns of MLL proteins at gene promoters in wt MLL-depleted cells. The distribution pattern of MLL ChIP signals around transcription start sites (TSSs) in HB1119 cells with or without knockdown of wt MLL was analyzed in a genome wide manner. The data are partially redundant with our previous report (Okuda et al., 2017).

**Figure 1—figure supplement 2.**
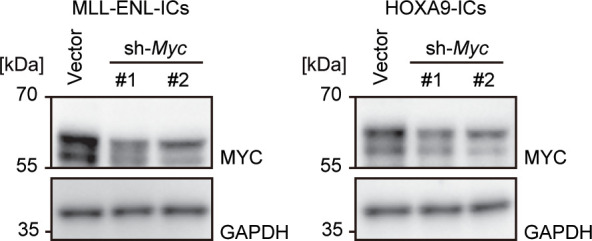
Expression of the MYC protein after *Myc* knockdown. Western blotting was performed on MLL-ENL- and HOXA9-ICs to demonstrate the knockdown effects of shRNA for *Myc*.

To characterize the dynamic changes in MLL target gene expression during differentiation, we isolated bone marrow cells from mice at various differentiation stages ranging from the most immature population (c-Kit^+^, Sca1^+^, Lineage^–^; KSL) containing hematopoietic stem cells to highly differentiated hematopoietic cells (c-Kit^low^/Mac1^high^) by fluorescence-activated cell sorting (FACS) and performed quantitative reverse transcription-polymerase chain reaction (qRT-PCR) analysis. For comparison, we analyzed leukemia cells (LCs) harvested from mice suffering with MLL-AF10-induced leukemia (MLL-AF10-LCs) and immortalized cells (ICs) transformed by MLL-ENL ex vivo (MLL-ENL-ICs). In accordance with previous reports (Krivtsov et al., 2006; Somervaille and Cleary, 2006; Yokoyama et al., 2013), the expression levels of *Hoxa9*, *Hoxa10*, and *Meis1* were downregulated during normal hematopoietic differentiation but remained high in MLL fusion-expressing cells (Figure 1B). *Myc* was highly expressed in KSL, common myeloid progenitors (CMPs), and granulocyte/macrophage progenitors (GMPs), which contain actively dividing populations (Passegué et al., 2005), but was completely suppressed at highly differentiated c-kit^low^/Mac1^high^ stages. In the two MLL fusion-expressing cell lines, *Myc* was expressed at comparable levels to those in the progenitor fractions (CMP and GMP; Figure 1B). *Mxd1*, a differentiation marker, was highly expressed in differentiated populations alone. These results indicate that *Myc*, *Hoxa9*, *Hoxa10*, and *Meis1* are intrinsically programmed to be silenced in normal hematopoietic differentiation but are aberrantly maintained by MLL fusion proteins.

To assess the oncogenic potential of MLL target genes, we performed myeloid progenitor transformation assays (Lavau et al., 1997; Okuda and Yokoyama, 2017a), wherein HPCs were isolated from mice, retrovirally transduced with each MLL target gene, and cultured in a semi-solid medium supplemented with cytokines promoting myeloid lineage differentiation (Figure 1C,D). HPCs exogenously expressing MLL-ENL or MLL-AF10 produced a large number of colonies in the third and fourth passages with high mRNA levels of *Myc*, *Hoxa9*, *Hoxa10*, and *Meis1* in colonies of the first passage, confirming their potent transforming capacities. These cells were considered ‘immortalized’ as they proliferate indefinitely in this ex vivo culture (Lavau et al., 1997; Okuda and Yokoyama, 2017a). Ectopic expression of MYC immortalized HPCs; therefore, MYC expression is sufficient to induce proliferation of HPCs. Endogenous *Myc* expression was severely diminished in MYC-ICs, demonstrating that *Myc* expression is programed to be silenced and cannot be sustained by MYC itself. Ectopic expression of HOXA9 and HOXA10 (but not MEIS1) immortalized HPCs. Endogenous *Myc* expression was maintained in HOXA9/A10-ICs, indicating that HOXA9/A10 can sustain *Myc* expression against differentiation-induced transcriptional suppression. It should be noted that the colony size of HOXA9/A10-ICs was relatively small compared with MYC- or MLL fusion-ICs, suggesting a weaker proliferative potential. Knockdown of *Myc* by shRNA completely repressed the colony-forming ability of MLL-ENL-ICs and HOXA9-ICs (Figure 1E and Figure 1—figure supplement 2). Taken together, these results demonstrate that MLL fusion proteins and HOXA9 maintain *Myc* expression despite of the differentiation-induced transcriptional suppression, and the maintenance of MYC expression is indispensable for immortalization of HPCs ex vivo.

### HOXA9 confers the identity of a hematopoietic precursor while MYC drives anabolic pathways

**Figure 2.**
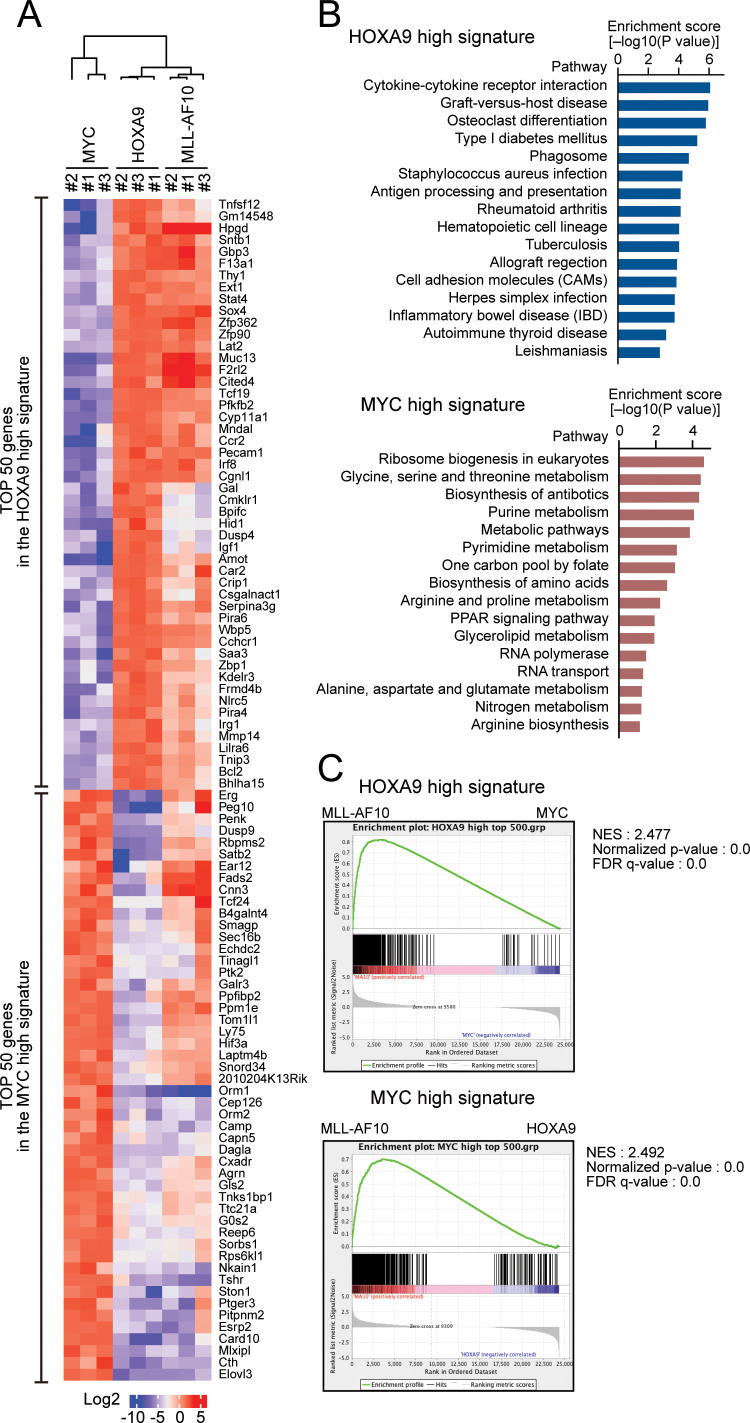
HOXA9 confers the identity of a hematopoietic precursor while MYC drives anabolic pathways. (**A**) Relative expression of the top 50 genes categorized as the HOXA9 high signature and the MYC high signature in HOXA9-, MYC-, and MLL-AF10-ICs. The RPKM data are provided in Figure 2—source data 1. (**B**) Pathways related to the HOXA9 high signature (blue) and the MYC high signature (red). The top 500 genes in the HOXA9 high or MYC high signatures were subjected to KEGG pathway analysis. The summary is provided in Figure 2—source data 1 (**C**) Gene set enrichment analysis of the HOXA9 high and MYC high signatures in MLL-AF10-ICs compared with MYC-ICs (top) and HOXA9-ICs (bottom), respectively. Figure 2—source data 1.Gene expression profiles of HOXA9-, MYC-, and MLL-AF10-transformed cells.

To identify the genes specifically regulated by HOXA9 but not by MYC, we performed RNA-seq analysis of HOXA9-ICs and MYC-ICs which do not express *HOXA9* (Figure 1C). Genes highly expressed in HOXA9-ICs but lowly expressed in MYC-ICs (defined as ‘HOXA9 high signature’) were associated with hematopoietic identity/functions. In contrast, genes highly expressed in MYC-ICs and lowly expressed in HOXA9-ICs (defined as ‘MYC high signature’) were associated with anabolic pathways involved in nucleotide/protein production (Figure 2A,B). Pathways involved in lipid metabolism were commonly upregulated in both HOXA9- and MYC-ICs compared with non-immortalized c-kit-positive HPCs (Figure 2—figure supplement 1A,B). MLL-AF10-ICs, which express endogenous *Hoxa9* and *Myc* at high levels (Figure 1C), expressed both HOXA9 high and MYC high signature genes (Figure 2A–C), indicating that MLL-AF10-ICs possess MYC-mediated highly proliferative potential and HOXA9-mediated hematopoietic identity. These differences between the HOXA9 high and MYC high signatures indicate that HOXA9 maintains the identity of a hematopoietic precursor, while MYC promotes proliferation by upregulating anabolic pathways.

**Figure 2—figure supplement 1.**
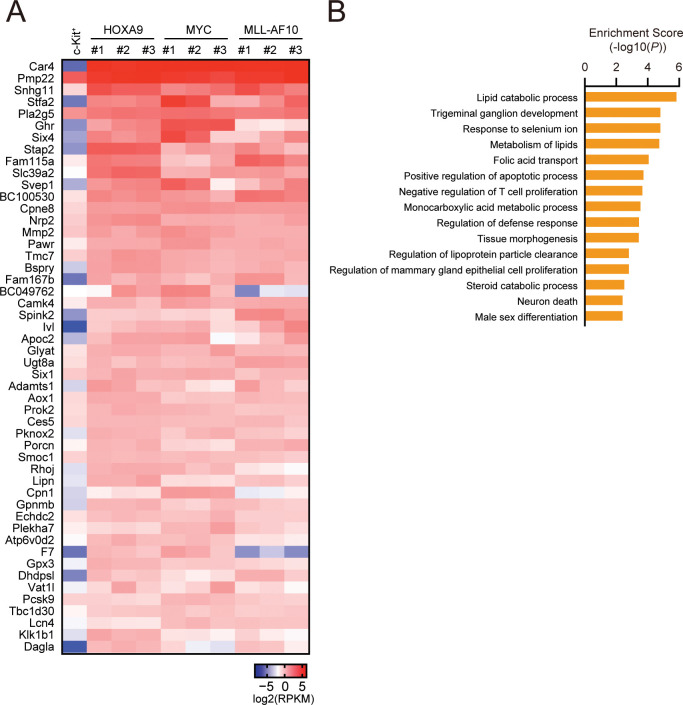
Biological pathways commonly regulated by HOXA9 and MYC. (**A**) Genes commonly regulated by HOXA9 and MYC. ICs used in Figure 2A and freshly prepared cKit+ cells were analyzed by RNA-seq. A representative set of commonly upregulated genes in HOXA9- and MYC-ICs compared to cKit+ cells are shown in a heatmap. (**B**) Pathways commonly regulated by HOXA9 and MYC. Genes exhibiting > 2-fold increase commonly in HOXA9- and MYC-ICs compared to c-Kit^+^ cells were grouped as ‘HOXA9-MYC common target genes’. Go pathway analysis for top 250 HOXA9-MYC common target genes was performed.

### Apoptosis is induced by MYC, and alleviated by HOXA9 and MLL-AF10

**Figure 3.**
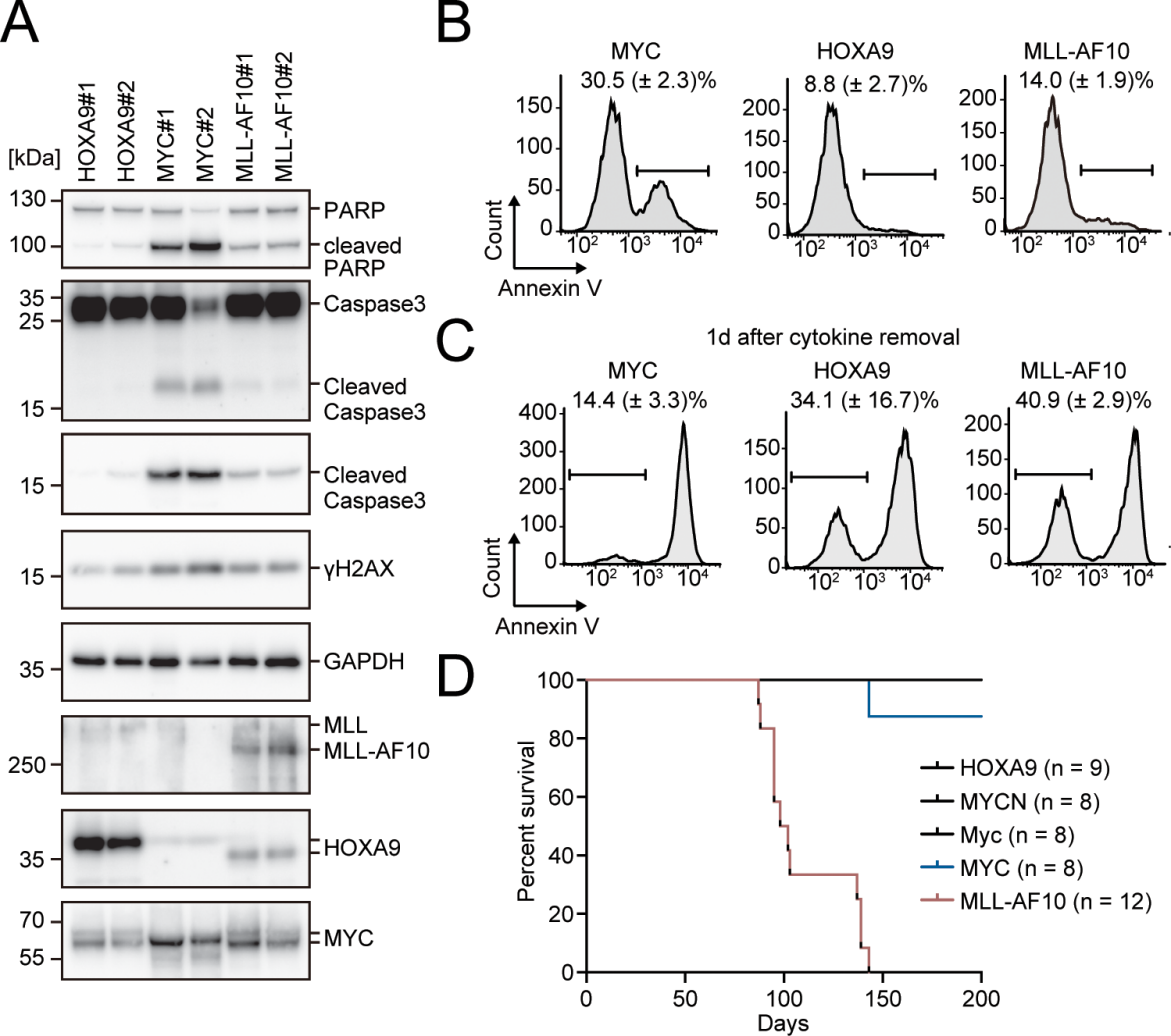
Apoptosis is induced by MYC, while is alleviated by HOXA9 and MLL-AF10. (**A**) Protein expression of transgenes and apoptotic markers in HOXA9-, MYC-, and MLL-AF10-ICs. (**B** and **C**) Apoptotic tendencies of HOXA9-, MYC-, and MLL-AF10-ICs. Representative FACS plots and the summarized data (Mean with SD, n = 3, biological replicates) of Annexin V staining of HOXA9-, MYC-, and MLL-AF10-ICs in the presence of cytokines (**B**) and 1d after their removal (**C**) are shown. (**D**) In vivo leukemogenic potential of MLL target genes and MLL-AF10. Kaplan-Meier curves of mice transplanted with HPCs transduced with the indicated genes and the number of replicates are shown.

Given that excessive MYC activity promotes apoptosis (McMahon, 2014), we evaluated the apoptotic tendencies in immortalized HPCs. MYC-ICs exhibited increased γH2AX, cleaved poly (ADP) ribose polymerase (PARP), and cleaved caspase 3 levels, indicative of a high degree of replication stress and apoptosis (Figure 3A). FACS analysis with Annexin V also showed that MYC-ICs had more significant apoptotic fraction than that of HOXA9-ICs (Figure 3B). MLL-AF10-ICs exhibited weak apoptotic tendencies similarly to HOXA9-ICs, although their MYC expression tended to be higher than HOXA9-ICs (Figures 1C and 3A). Furthermore, most MYC-ICs underwent massive apoptosis 1 day after cytokine removal, whereas HOXA9-ICs and MLL-AF10-ICs showed relative resistance (Figure 3C) and exhibited successful recovery of the live cell population after cytokine reintroduction (Figure 3—figure supplement 1). These results indicate that HOXA9 confers anti-apoptotic properties.

**Figure 3—figure supplement 1.**
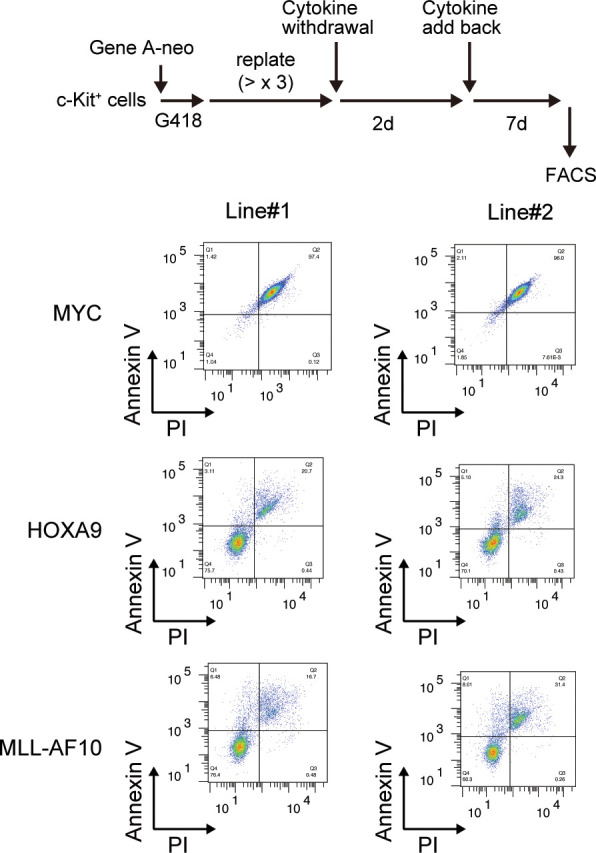
| Survival of HOXA9-expressing cells upon cytokine withdrawal. MYC-, HOXA9-, MLL-AF10-ICs were subjected to cytokine withdrawal for 2 days, then cultured in the presence of cytokines for 7 days, and examined by FACS for Annexin V- and Propidium Iodide (PI)-positivity.

Next, we evaluated the leukemogenic potential of HOXA9 and MYC in vivo. Transplantation of HPCs exogenously expressing MLL-AF10 into syngeneic mice induced leukemia with full penetrance (Figure 3D). In contrast, neither *MYC* nor its homologue *MYCN* could initiate leukemia within 200 days under these experimental conditions, indicating that activation of the MYC high signature alone is insufficient for leukemogenesis in vivo. Although one recipient mouse of *MYC*-transduced HPCs became sick and was sacrificed approximately 150 days after transplantation, it did not exhibit any leukemia-associated signs. The mouse *Myc* gene also failed to induce leukemia. Thus, it is unlikely that the inability of MYC alone to induce leukemia under these experimental conditions is due to immune suppression of the cells expressing human transgenes. *HOXA9* did not induce leukemia within 200 days either, suggesting that the HOXA9 high signature alone is also insufficient to induce leukemia. Taken together, these results suggest that both high MYC activity and HOXA9-mediated resistance to apoptosis are necessary for driving leukemogenesis in vivo.

### HOXA9 promotes MYC-mediated leukemogenesis

**Figure 4.**
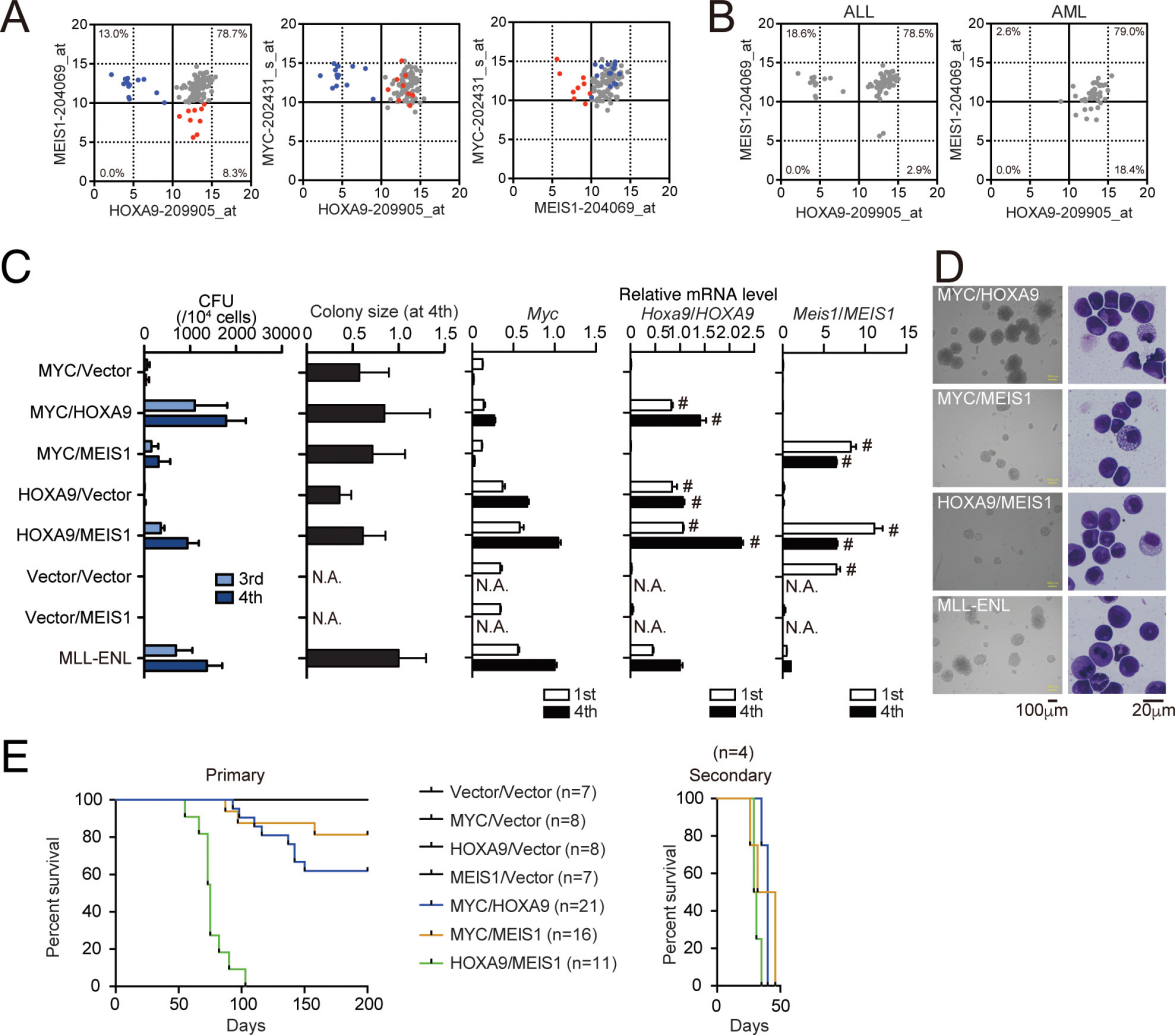
HOXA9 promotes MYC-mediated leukemogenesis. (**A** and **B**) Expression profiles of MLL fusion-mediated leukemia patients reported in the MILE study (Haferlach et al., 2010). Probe intensities of the indicated genes are plotted for all MLL fusion-mediated leukemia patients (**A**). Patients in the HOXA9^low^/MEIS1^high^ (blue) and HOXA9^high^/MEIS1^low^ (red) groups are highlighted. Probe intensities of *HOXA9* and *MEIS1* are plotted separately by leukemia phenotype (ALL or AML) (**B**). The expression profiles are provided in Figure 4—source data 1. (**C**) Transforming potential of various combinations of MLL target genes. CFU (Mean with SD, n = 3, biological replicates) and relative colony size (Mean with SD, n ≥ 100) are shown as in Figure 1C. (**D**) Morphologies of the colonies and transformed cells. Bright-field (left) and May-Grunwald-Giemsa staining (right) images are shown with scale bars. (**E**) In vivo leukemogenic potential of various oncogene combinations. Kaplan-Meier curves of mice transplanted with HPCs transduced with the indicated genes are shown as in Figure 3D. Bone marrow cells from moribund mice were harvested and used for secondary transplantation. Figure 4—source data 1.Gene expression profiles of MLL fusion-mediated leukemia patients in the MILE data.

We next examined the gene expression of patients with leukemia using publicly available microarray data of the Microarray Innovations in LEukemia (MILE) study (Haferlach et al., 2010). Of 108 cases with MLL translocation, 38 were acute myelogenous leukemia [AML], and 70 were acute lymphoblastic leukemia [ALL]. Most of the cases (78.7%) were categorized in the HOXA9^high^/MEIS1^high^ group, while some were in the HOXA9^low^/MEIS1^high^ (13.0%) or HOXA9^high^/MEIS1^low^ (8.3%) group (Figure 4A). *MYC* was expressed at high levels irrespective of *HOXA9* or *MEIS1* expression. These data indicate variability in transcriptional profiles among MLL fusion-mediated leukemia cases, with *MYC* expression remaining consistently high. HOXA9^low^/MEIS1^high^ leukemia was predominantly found in ALL, likely due to the MLL-AF4 cases, some of which do not express *HOXA* genes (Lin et al., 2016). HOXA9^high^/MEIS1^low^ leukemia was mainly found in AML (Figure 4B).

**Figure 4—figure supplement 1.**
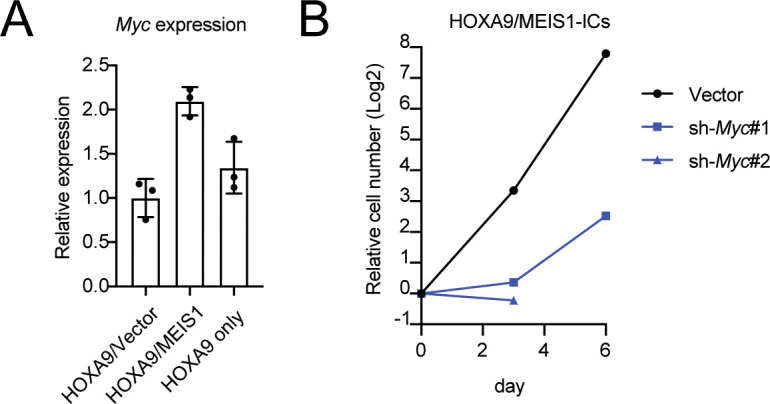
Role of MYC in HOXA9/MEIS1-mediated transformation. (**A**) *Myc* expression levels in HOXA9- and HOXA9/MEIS1-transduced cells. *Myc* RNA levels of three biological replicates of indicated ICs were determined by qRT-PCR (Mean with SD). (**B**) Inhibition of the proliferation of HOXA9/MEIS1-transduced cells by *Myc* knockdown. The same shRNA used in Figure 1E were transduced to HOXA9/MEIS-ICs and their cell number was counted every 3 days.

**Figure 4—figure supplement 2.**
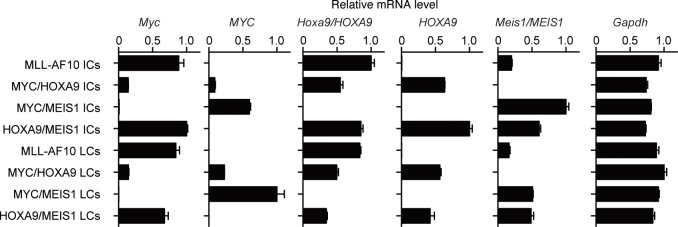
Expression of endogenous *Myc* and the transgenes in immortalized- and leukemia-cells. Relative mRNA levels of the indicated genes (Mean with SD, n=3, PCR replicates) are shown along with those of the respective ICs. LCs were cultured ex vivo for several passages to remove non-leukemia cells and subjected to qRT-PCR.

**Figure 4—figure supplement 3.**
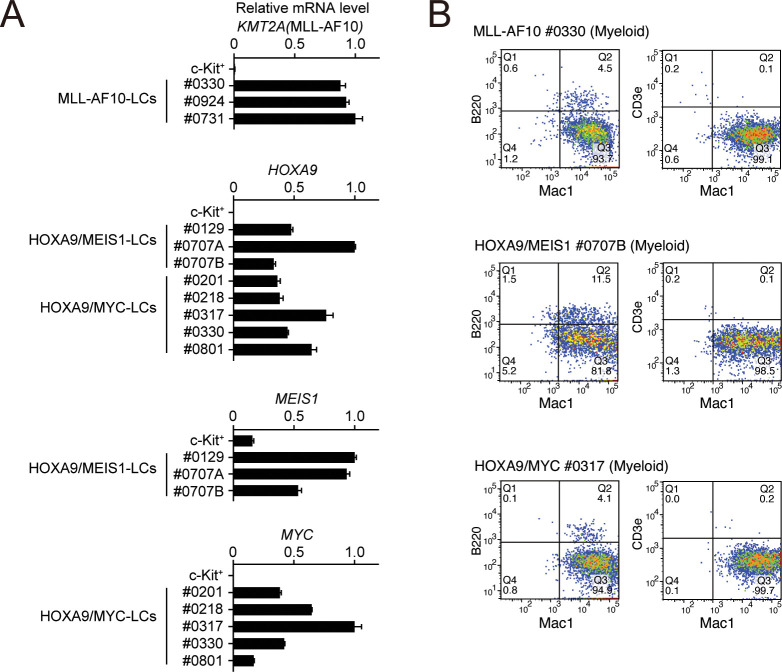
Phenotypes of MLL-AF10-, HOXA9/MEIS1-, and HOXA9/MYC-mediated leukemia cells at the endpoint. (**A**) Expressions of transgenes at the endpoint in the LCs described in Figure 4E. Cells were harvested from moribund mice and were analyzed by qRT-PCR (Mean with SD, n = 3, PCR replicates). cDNA of c-Kit^+^ cells were used as negative control. (**B**) Immunophenotype of the leukemia cells in Figure 4E. Cells were harvested from moribund mice and were analyzed by FACS using indicated antibodies.

**Figure 4—figure supplement 4.**
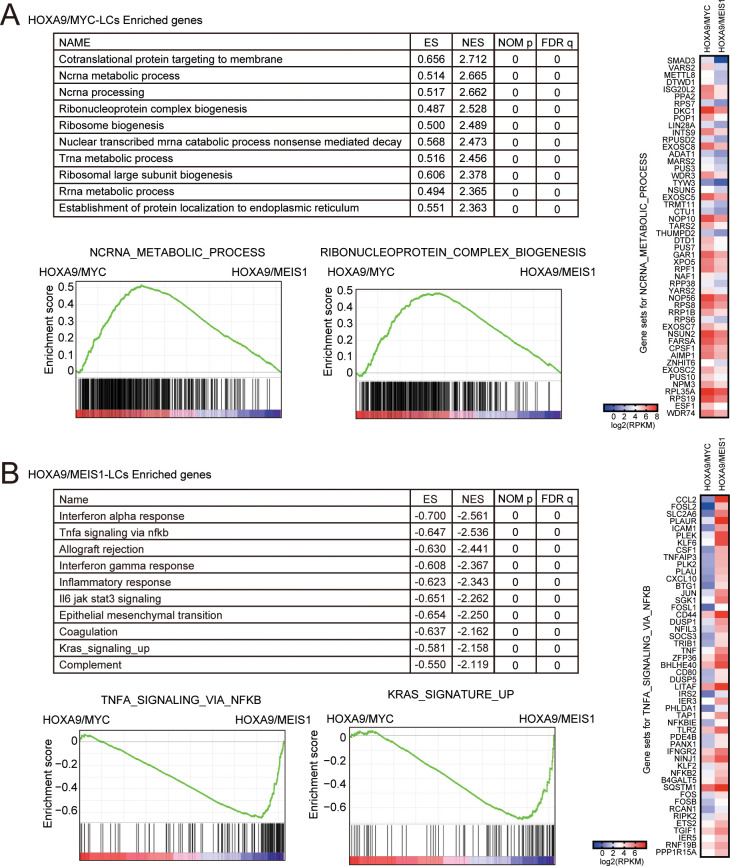
Biological pathways differentially regulated in HOXA9/MYC- and HOXA9/MEIS1-driven leukemias. (**A**) Pathways upregulated uniquely by HOXA9/MYC compared to HOXA9/MEIS1. Gene set enrichment analysis for HOXA9/MYC-LCs was performed against HOXA9/MEIS1-LCs. Ten representative enriched pathways and two of their enrichment curves are shown. A heatmap of the expression levels of ‘ncRNA metabolic process’ genes is shown on the right. (**B**) Pathways upregulated uniquely by HOXA9/MEIS1 compared to HOXA9/MYC. Gene set enrichment analysis is performed as in A. A heatmap of the expression levels of ‘TNFα signaling via NFκB’ genes is shown on the right.

Next, we assessed the effects of the combined expression of MYC, HOXA9, and MEIS1 in mouse leukemia models. The MYC/HOXA9 combination exhibited high clonogenicity ex vivo, with colony-forming capacity and colony size comparable to those of MLL-ENL- and HOXA9/MEIS1-ICs (Figure 4C,D). A weak synergy between MYC and MEIS1 was also observed. HOXA9/MEIS1-ICs showed high *Myc* expression (Figure 4—figure supplement 1A) and MYC-dependent proliferation (Figure 4—figure supplement 1B), indicating that MYC is essential in HOXA9/MEIS1-mediated leukemic transformation. In the in vivo leukemogenesis assays, MYC, HOXA9, or MEIS1 expression alone did not initiate leukemia, whereas co-expression of HOXA9 and MEIS1 did in all recipient mice as previously reported (Figure 4E; Kroon et al., 1998). The combined expression of MYC/HOXA9 or MYC/MEIS1 induced leukemia in 38% and 18% of recipient mice, respectively, indicating a synergy of MYC with HOXA9 and MEIS1. qRT-PCR analysis showed that LCs maintained the expression patterns of endogenous *Hoxa9*, *Meis1*, and *Myc* similar to those of the respective ICs (Figure 4—figure supplement 2). We observed rapid onset of leukemia with full penetrance in the secondary transplantation for the three combinations tested, confirming the presence of leukemia-initiating cells (Figure 4E). These results indicate a cooperative role for HOXA9 and MEIS1 in MYC-mediated leukemogenesis with stronger synergy between HOXA9 and MYC.

All the MYC/HOXA9- and HOXA9/MEIS1-induced leukemias were positive to a myeloid marker Mac1 but negative to lymphoid marker B220 or CD3e (Figure 4—figure supplement 3A,B), indicating that both MYC/HOXA9- and HOXA9/MEIS1-combinations induced myeloid leukemia. Gene set enrichment analysis (GSEA) of MYC/HOXA9-LCs and HOXA9/MEIS1-LCs revealed that genes involved in protein synthesis (e.g. ribosome biogenesis, tRNA metabolic process) were enriched in MYC/HOXA9-LCs (Figure 4—figure supplement 4A), while tumour necrosis factor α signaling and KRAS pathways were enriched in HOXA9/MEIS1-LCs (Figure 4—figure supplement 4B). These results suggested that these two combinations promoted leukemogenesis in slightly different ways, wherein MYC-mediated anabolic pathways played more prominent roles in leukemogenesis by MYC/HOXA9 than by HOXA9/MEIS1.

### HOXA9 functions as a transcription maintenance factor

**Figure 5.**
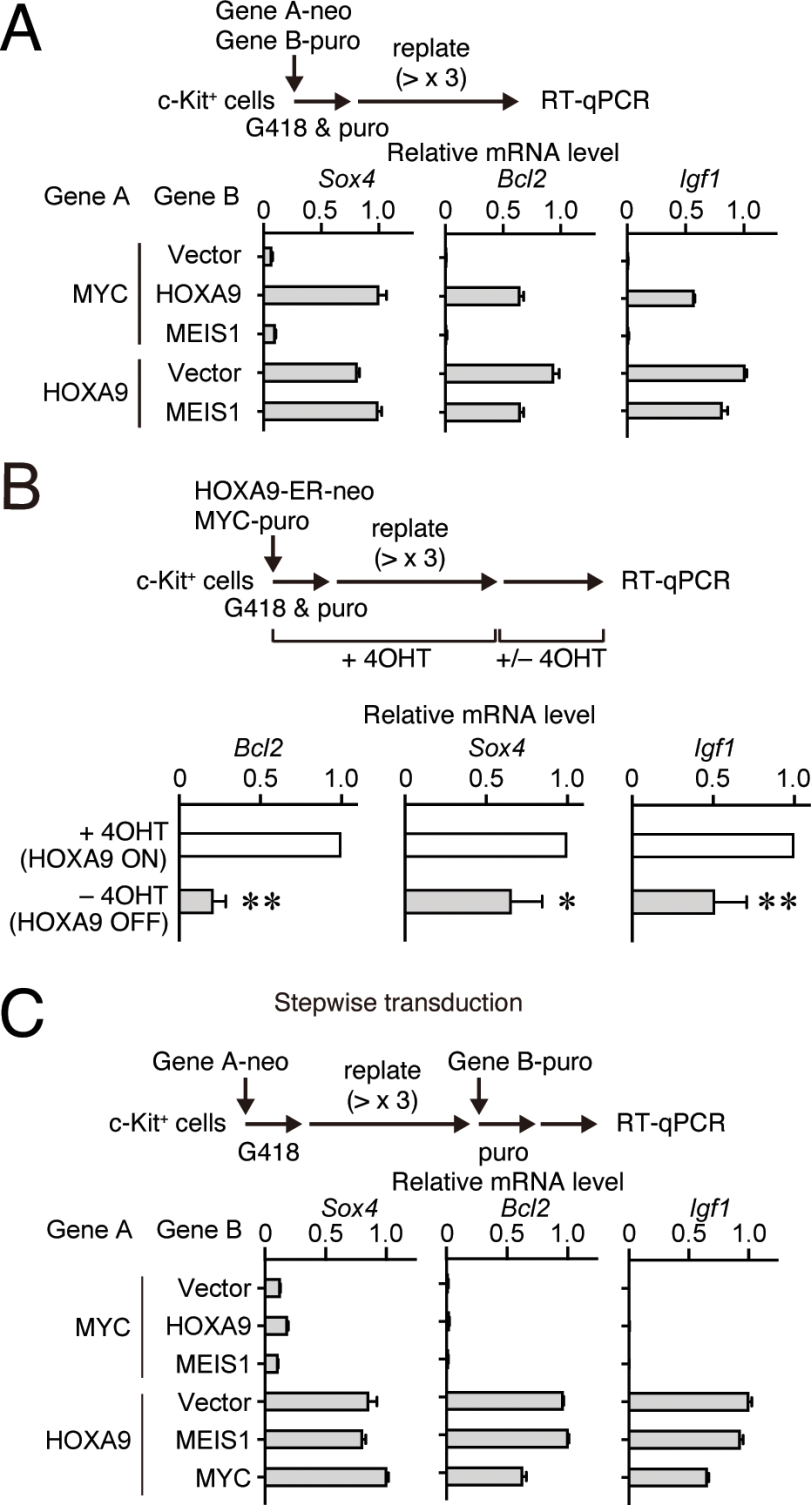
HOXA9 functions as a transcription maintenance factor. (**A**) Gene expression of HPCs immortalized by various transgenes. Relative mRNA levels of HOXA9 target genes (Mean with SD, n = 3, PCR replicates) in myeloid progenitors transformed by various combinations of MLL target genes are shown. Two genes were transduced into HPCs in a simultaneous manner. (**B**) Gene expression after inactivation of HOXA9. HOXA9-ER and MYC were doubly transduced into HPCs and cultured in the presence of 4-OHT ex vivo. After 4-OHT withdrawal, qRT-PCR analysis was performed for the indicated genes (Mean, n=4, biological replicates). Statistical analysis was performed using unpaired two-tailed Student’s *t*-test. **p< 0.01, *p < 0.05. (**C**) Gene expression of HPCs immortalized by step-wise transduction of various transgenes. Relative mRNA levels of HOXA9 target genes in myeloid progenitors transformed by various combinations of MLL target genes are shown as in A. Two genes were transduced into HPCs in a stepwise manner.

Some HOXA9 high signature genes, namely *Bcl2*, *Sox4*, and *Igf1*, have been implicated in leukemogenesis (Zhang et al., 2013; Du et al., 2005; Delbridge et al., 2016; Steger et al., 2015), suggesting that they may be responsible for the synergy between HOXA9 and MYC. Indeed, *Bcl2, Sox4*, and *Igf1* were highly transcribed in HPCs transformed by the combinations containing HOXA9 (i.e. MYC/HOXA9-ICs, HOXA9/vector-ICs, and HOXA9/MEIS1-ICs) but not those lacking it (i.e. MYC/vector-ICs and MYC/MEIS1-ICs) (Figure 5A and Figure 5—figure supplement 1A). Conditional loss of function experiments using HOXA9 conjugated with estrogen receptor (HOXA9-ER) confirmed critical regulation of these genes by HOXA9 (Figure 5B). However, stepwise transduction of *MYC* followed by *HOXA9* failed to upregulate these HOXA9 target genes, indicating that HOXA9 could not reactivate its target genes once silenced (Figure 5C and Figure 5—figure supplement 1B). Conditional inactivation and subsequent reactivation of HOXA9 within the same cell population confirmed that HOXA9 cannot reactivate its target genes once silenced (Figure 5—figure supplement 1C). RNA-seq analysis of various human cell lines showed that *HOXA9* was highly expressed in all the MLL fusion cell lines tested (Figure 5—figure supplement 2A, highlighted in blue). *BCL2* and *SOX4* were also expressed but at different levels depending on the cell lines. In accord, most of the MLL fusion-mediated leukemia patient samples in the MILE study with high *HOXA9* expression expressed *BCL2* and *SOX4* at high levels (Figure 5—figure supplement 2B; Haferlach et al., 2010). *IGF1* was only expressed in MV4-11 cells despite comparable *HOXA9* expression among the MLL fusion cell lines, supporting the notion that HOXA9 expression itself cannot trigger the expression of silenced HOXA9 target genes. These results indicate that HOXA9 is a transcriptional maintenance factor that may be involved in the maintenance of chromatin structure previously activated by other transcriptional/epigenetic factors.

**Figure 5—figure supplement 1.**
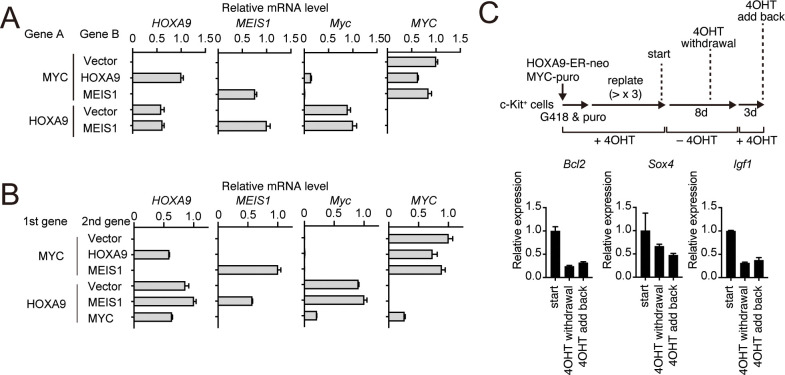
HOXA9-dependent gene expression of various HOXA9 target genes. (**A**) Relative mRNA levels of the transgenes and endogenous *Myc* in the cells used in Figure 5A. (**B**) Relative mRNA levels of the transgenes and endogenous *Myc* in the cells used in Figure 5C. (**C**) Expression of HOXA9 target genes during the inactivation and subsequent reactivation of HOXA9. Schema is shown on top. qRT-PCR was performed at the indicated time point (Mean with SD, n = 3, PCR replicates).

**Figure 5—figure supplement 2.**
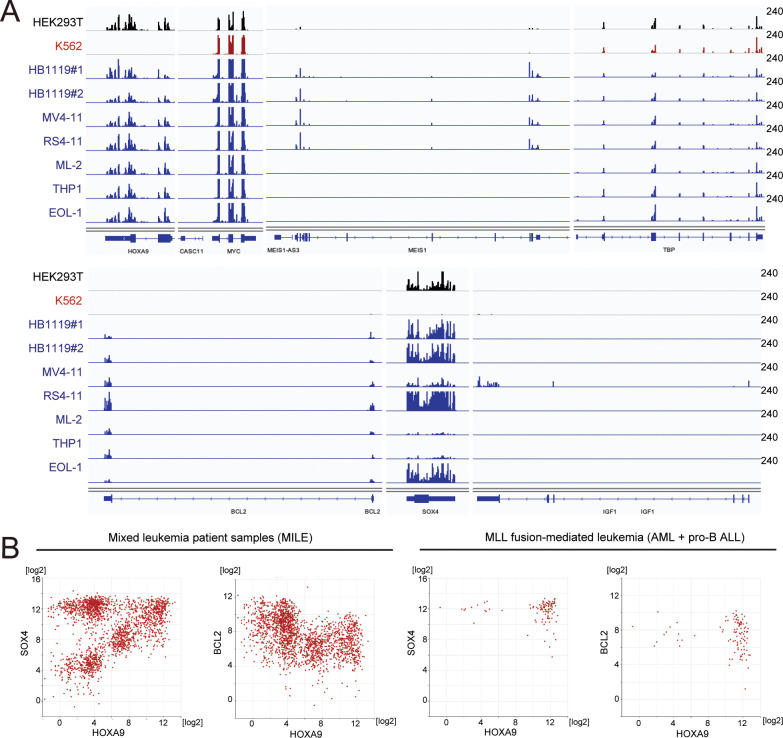
Gene expression of various HOXA9 target genes in cell lines and patients. (**A**) RNA-seq analysis of various MLL fusion cell lines (highlighted in blue). Non-MLL fusion cell lines (i.e. HEK293T and K562) were included for comparison. HB1119 cells were analyzed in duplicates. (**B**) Relative expression of HOXA9 and its target genes in leukemia patients of the MILE study (Haferlach et al., 2010). Relative expression levels of HOXA9 compared with SOX4 or BCL2 are shown in all of the mixed leukemia samples (left) and MLL fusion-mediated leukemia (AML and pro-B ALL) samples (right).

### BCL2 and SOX4 promote MYC-mediated leukemogenesis by alleviating apoptosis

**Figure 6.**
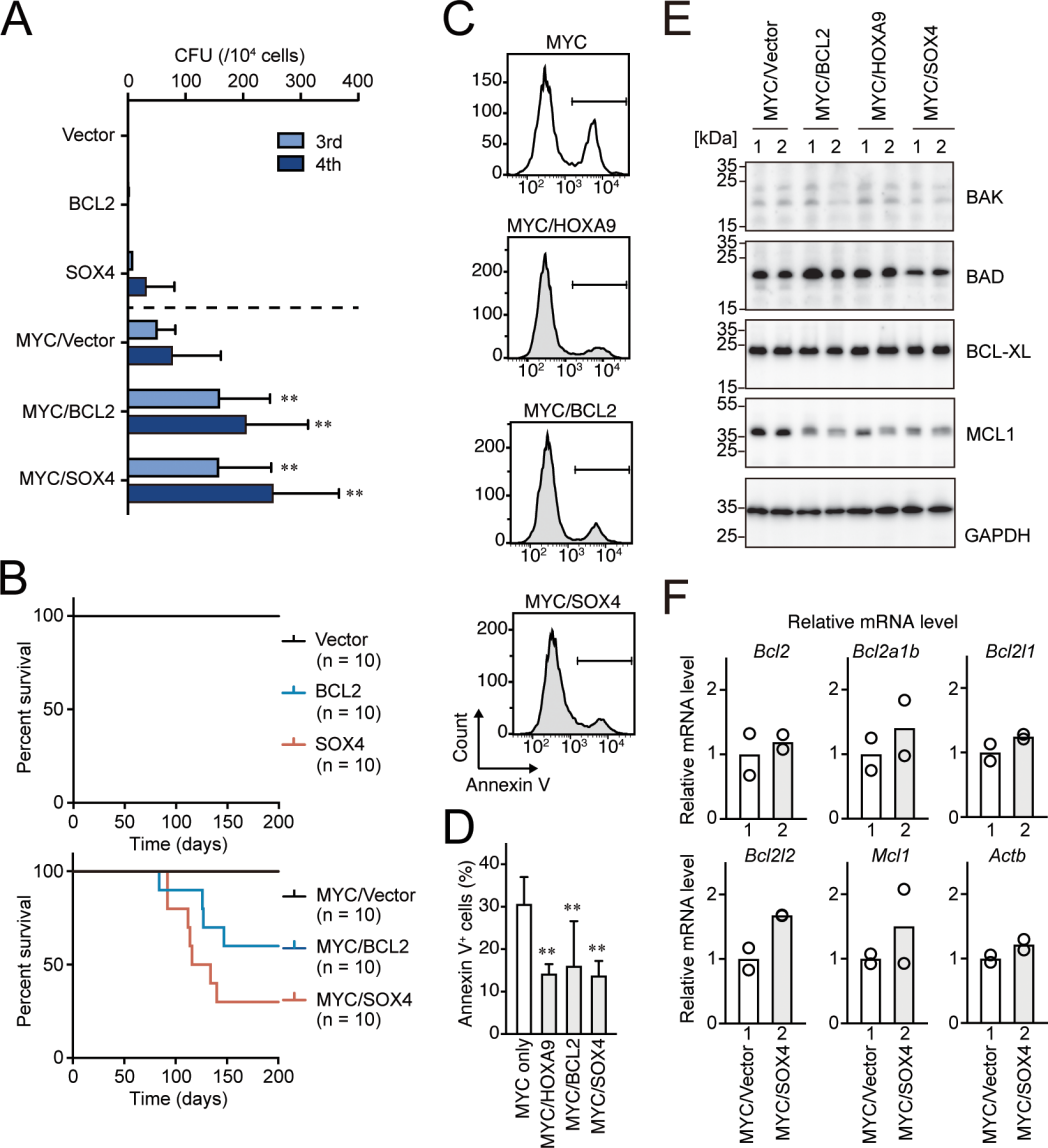
BCL2 and SOX4 promote MYC-mediated leukemogenesis by alleviating apoptosis. (**A**) Transforming potential of various combinations of MYC and HOXA9 target genes. CFU (Mean with SD, n = 3, biological replicates) is shown as in Figure 1C. (**B**) In vivo leukemogenic potential of various combinations of MYC and HOXA9 target genes. Kaplan-Meier curves of mice transplanted with HPCs transduced with the indicated genes are shown as in Figure 3D. (**C** and **D**) Apoptotic tendencies of *MYC*-expressing progenitors co-transduced with *HOXA9*, *BCL2*, or *SOX4*. Representative FACS plots (**C**) and the summarized data (**D**) (Mean with SD, n = 3, biological replicates) of Annexin V staining are shown. Statistical analysis was performed using ordinary one-way ANOVA with MYC-ICs. (**E**) Expression of apoptosis-related proteins in HPCs transformed by various combinations of transgenes. Western blots of HPCs transformed by indicated transgenes are shown (**F**) Relative expression levels of apoptosis-related genes in MYC/SOX4-ICs and MYC/vector-ICs. Relative mRNA levels of the indicated apoptosis-related genes (Mean, n = 2, biological replicates) are shown.

To identify the roles for BCL2 and SOX4 in leukemic transformation, we evaluated the leukemogenic potential of the combined expression of BCL2 or SOX4 with MYC. In myeloid progenitor transformation assays, SOX4 by itself showed weak immortalization capacity as previously reported (Zhang et al., 2013), while BCL2 did not transform HPCs (Figure 6A). Co-expression of BCL2 or SOX4 with MYC led to a substantial increase in colony-forming capacity. The combined expression of BCL2 with MYC induced leukemia in vivo with a penetrance similar to that with the MYC/HOXA9 combination (Figures 4E and 6B, and Figure 6—figure supplement 1A), in accord with previous reports (Beverly and Varmus, 2009; Luo et al., 2005). SOX4 also promoted MYC-mediated leukemogenesis in vivo (Figure 6B and Figure 6—figure supplement 1A), while neither MYC, BCL2, nor SOX4 alone induced leukemia within 200 days. FACS analysis indicated that all of MYC/SOX4-induced leukemias were of myeloid lineage (Figure 6—figure supplement 1B). In contrast, all MYC/BCL2-induced leukemias were of lymphoid lineage, the half of which was B-cell type, and the other half was T-cell type, consistent with a previous report (Luo et al., 2005). It should be noted that single gene transduction of MYC and SOX4 induced leukemia by others in different settings, albeit with low penetrance (Du et al., 2005; Luo et al., 2005). These differences are possibly due to the differences of virus titers and viral genome integration-related gene activation. Furthermore, the combined expression of HOXA9, BCL2, or SOX4 with MYC alleviated apoptotic tendencies (Figure 6C,D). Although SOX4 is reported to modulate transcription of pro/anti-apoptotic genes (Ramezani-Rad et al., 2013), their expression was not drastically altered by SOX4 in this context (Figure 6E,F). Thus, the mechanism underlying the anti-apoptotic properties of SOX4 in this setting is currently unclear. Taken together, the results indicate that multiple anti-apoptotic pathways mediated by BCL2 and SOX4 promote MYC-mediated leukemic transformation.

**Figure 6—figure supplement 1.**
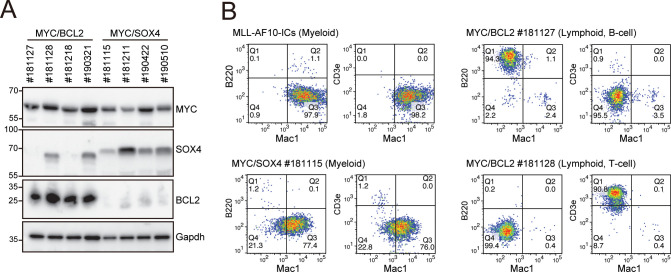
Phenotypes of MYC/BCL2- and MYC/SOX4-mediated leukemias at the endpoint. (**A**) Expression of the transgenes in LCs induced by various combinations of transgenes. LCs were harvested from moribund mice (n = four each) and analyzed by western blotting. (**B**) Immunophenotype of LCs induced by various combinations of transgenes. Cells were harvested from moribund mice and were analyzed by FACS using indicated antibodies.

### Endogenous BCL2 and SOX4 support the initiation and maintenance of leukemia

**Figure 7.**
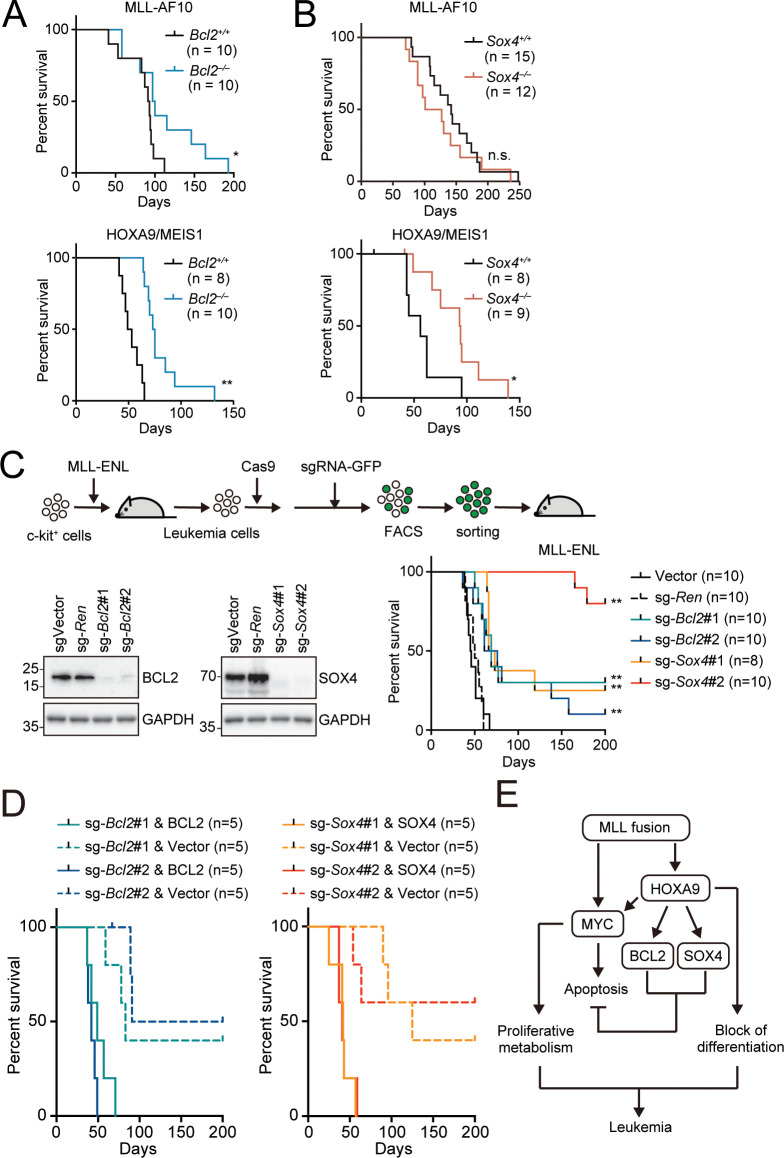
Endogenous BCL2 and SOX4 support the initiation and maintenance of leukemia. (**A**) Effects of *Bcl2*-deficiency on the initiation of leukemogenesis in vivo. HPCs were isolated from *Bcl2*^+/+^ or *Bcl2*^–/–^ embryos and transduced with the retroviruses for MLL-AF10 or the HOXA9/MEIS1 combination. Kaplan-Meier curves of mice transplanted with the transduced HPCs are shown. Statistical analysis was performed using the log-rank test and Bonferroni correction with the wildtype control. *p ≤ 0.05. (**B**) Effects of *Sox4*-deficiency on the initiation of leukemogenesis in vivo. In vivo leukemogenesis assay was performed on *Sox4*^+/+^ or *Sox4*^–/–^ embryos as in A (**C**) Effects of *Bcl2*- or *Sox4*-deficiency on the maintenance of leukemia initiating cells. Western blots of MLL-ENL leukemia cells transduced with sgRNA for BCL2 or SOX4 are shown on the left. Kaplan-Meier curves of mice transplanted with MLL-ENL leukemia cells transduced with the indicated sgRNAs are shown on the right. Statistical analysis was performed using the log-rank test and Bonferroni correction with the vector control. (**D**) Rescue of in vivo leukemogenic potential by sgRNA-resistant transgenes. Before transduction of sgRNA, MLL-ENL-LCs were transduced with sgRNA-resistant *BCL2* or *SOX4*. In vivo leukemogenesis assay was performed as described in Figure 3D. (**E**) A model illustrating HOXA9-mediated pathogenesis in MLL fusion-mediated leukemia.

To examine the roles for endogenous BCL2 and SOX4 in the initiation and maintenance of LCs, we conducted myeloid progenitor transformation and in vivo leukemogenesis assays using *Bcl2*- and *Sox4*-knockout HPCs. We transduced various oncogenes into HPCs isolated from fetal livers of *Bcl2*- and *Sox4*-knockout embryos (Kamada et al., 1995; Schilham et al., 1996) and cultured them ex vivo. Neither *Bcl2* nor *Sox4* deletion affected the immortalization of HPCs by HOXA9, MYC, or MLL-AF10, indicating that BCL2 and SOX4 are dispensable for proliferation ex vivo (Figure 7—figure supplement 1). On the other hand, *Bcl2* deletion delayed the onset of leukemia induced by MLL-AF10 and the HOXA9/MEIS1 combination in vivo (Figure 7A and Figure 7—figure supplement 2A). *Sox4* deletion also delayed the onset of HOXA9/MEIS1-induced leukemia, although it did not affect MLL-AF10-induced leukemia (Figure 7B and Figure 7—figure supplement 2A). We also analyzed the steady-state apoptosis of LCs harvested from moribund mice with apoptotic markers (cleaved caspase, γH2AX and Annexin V). HOXA9/MEIS1-LCs were slightly more apoptotic in the absence of *Bcl2* and *Sox4* (Figure 7—figure supplement 2B,C). However, we did not observe substantial differences in MLL-AF10-LCs between the WT and *Bcl2*/*Sox4* KO genotypes. These results are consistent with the different kinetics of leukemia onset (Figure 7A,B), and the minor differences in apoptotic tendencies in full-blown leukemia cells suggest that these LCs had acquired adequate anti-apoptotic properties at the time of disease presentation. These results indicate that endogenous BCL2 and SOX4 partially contribute to initiating leukemia in vivo.

**Figure 7—figure supplement 1.**
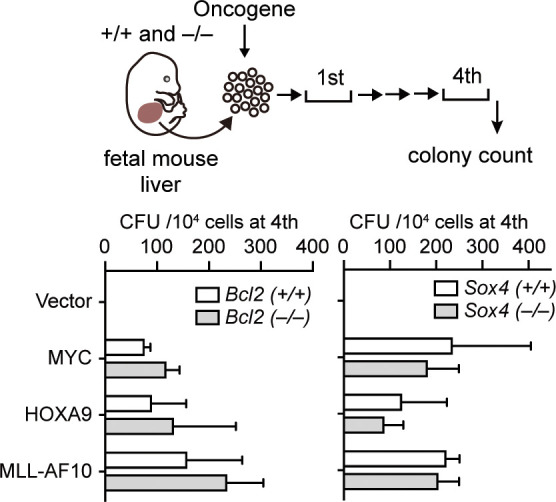
Requirement of BCL2 and SOX4 in transformation ex vivo. Retroviruses for MLL-AF10, HOXA9, or MYC were transduced into HPCs isolated from fetal liver of the indicated genotypes. CFU (Mean with SD, n = 3, biological replicates) of myeloid progenitors derived from *Bcl2*- or *Sox4*-knockout embryos is shown.

**Figure 7—figure supplement 2.**
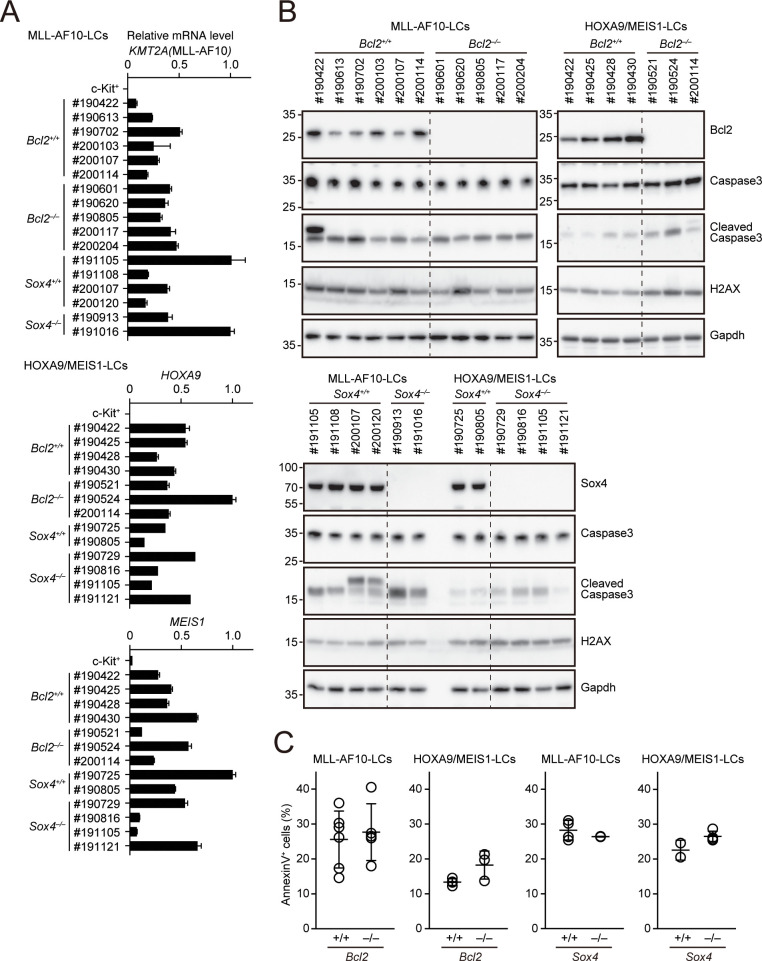
Apoptotic tendencies of Bcl2- or Sox4-deficient leukemia cells. (**A**) Expressions of transgenes in LCs. Leukemia cells were harvested from moribund mice. (MLL-AF10; *Bcl2^+/+^*, n = 6, MLL-AF10; *Bcl2^–/–^*, n = 5, MLL-AF10; *Sox4^+/+^*, n = 4, MLL-AF10; *Sox4^–/–^*, n = 2, HOXA9/MEIS1; *Bcl2^+/+^*, n = 4, HOXA9/MEIS1; *Bcl2^–/–^*, n = 3, HOXA9/MEIS1; *Sox4^+/+^*, n = 2, HOXA9/MEIS1; *Sox4^–/–^* n = 4,). Cells were harvested from moribund mice and were analyzed by qRT-PCR (Mean with SD, n = 3, PCR replicates). cDNA of c-Kit^+^ cells were used as negative control. (**B**) Expressions of apoptotic markers in LCs. Cleaved caspase three and γH2AX were analyzed by western blotting. (**C**) Apoptotic tendencies of LCs. AnnexinV positivity was analyzed by FACS as in Figure 3B.

**Figure 7—figure supplement 3.**
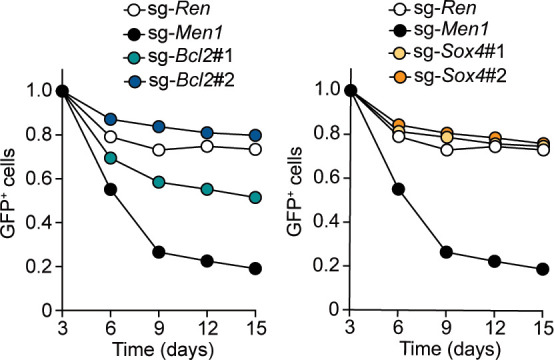
Effects of *Bcl2* or *Sox4* knockout on MLL-ENL-leukemia cells. sgRNA/GFP was transduced into Cas9-expressing MLL-ENL-LCs. Changes in GFP^+^ cells (%) (Mean n=3, biological replicates) are shown with sg-*Men1* serving as a positive control.

Next, we examined the roles for endogenous BCL2 and SOX4 in maintaining leukemia-initiating cells using a mouse leukemia cell line that we previously established with MLL-ENL (Okuda et al., 2017). CRISPR/Cas9-mediated sgRNA competition assays indicated the negligible contribution of *Bcl2* and *Sox4* to MLL-ENL-LC proliferation ex vivo (Figure 7—figure supplement 3). To test their roles in vivo, we transplanted *Bcl2*- or *Sox4*-deficient LCs into syngeneic mice, where we observed delayed onset and reduced incidence rate for both *Bcl2*- and *Sox4*-knockout LCs (Figure 7C). Forced expression of sgRNA-resistant cDNAs of *BCL2* and *SOX4* restored leukemogenic potential, validating the on-target effects of sgRNAs (Figure 7D). These results suggest that MLL-ENL-LCs are partially dependent on endogenous BCL2 and SOX4 in vivo. Taken together, our results indicate that MLL fusion-mediated LCs depend on the expression of multiple anti-apoptotic genes via HOXA9 to varying degrees to achieve survival advantages for disease initiation and maintenance (Figure 7E).

## Discussion

In this study, we found that HOXA9 regulates a variety of genes to maintain hematopoietic precursor identity and its associated anti-apoptotic properties. In leukemic transformation, MLL fusion proteins exploit both HOXA9 and MYC downstream pathways. Accordingly, HOXA9 and MYC synergistically induce leukemia in mouse models. Thus, we propose that MLL fusion proteins employ two arms to promote oncogenesis: MYC-mediated proliferation and HOXA9-mediated resistance to differentiation/apoptosis.

It is widely accepted that two types of mutations need to occur before leukemia onset; class I mutations that confer proliferative advantages and class II mutations that block differentiation (Gilliland, 2002). However, it was unclear how MLL mutations fit this theory because MLL fusion-mediated leukemia does not require additional mutations in many cases (Andersson et al., 2015). A comparison of gene expression profiles of HOXA9- and MYC-transformed HPCs demonstrated that HOXA9 maintains the expression of a wide range of genes associated with hematopoietic precursor identity. Thus, HOXA9 appears to maintain the intrinsic transcriptional programs of immature HPCs, which are programed to be silenced during differentiation. On the other hand, MYC upregulates genes involved with de novo nucleotide/protein synthesis. This is in line with the known involvement of MYC in proliferation and associated anabolic processes (Ji et al., 2011). Furthermore, MLL-AF10 activated both the HOXA9 high and MYC high signature genes to induce leukemia while ectopic expression of HOXA9 and MYC synergistically induced leukemia. Thus, our findings suggest that MLL fusion proteins activate both HOXA9- and MYC-dependent programs as alternative mechanisms to the combination of class I and II mutations.

Although HOXA9 maintained MYC expression to immortalize myeloid progenitors ex vivo, it did not induce leukemia in vivo. We speculate that HOXA9 alone cannot maintain MYC expression at a level sufficient to confer leukemogenic ability in vivo. MLL fusion- and HOXA9/MEIS1-transduced cells, which are capable of inducing leukemia in vivo, expressed *Myc* 20–100% more than HOXA9-ICs (Figure 1C and Figure 4—figure supplement 1A). This additional MYC activity may be required to achieve sufficient leukemogenic ability, therefore MYC/HOXA9-transduced cells were able to induce leukemia in vivo (Figure 4E). It should be noted that MYC/HOXA9-ICs and -LCs expressed endogenous *Myc* at a lower level than other HOXA9-expressing cells (i.e. MLL-AF10- and HOXA9/MEIS1-ICs/LCs) (Figure 4—figure supplement 2), indicating that there is a threshold of MYC expression a cell can endure. We speculate that HOXA9 uplifts the threshold of MYC expression by conferring anti-apoptotic properties but cannot hyper-activate MYC expression by itself. Thus, additional MYC activation mediated by MLL fusions and MEIS1 confers proliferative advantages sufficient to induce leukemia in vivo.

The therapeutic efficacy of BCL2 inhibitor has been reported in AML including MLL fusion-mediated leukemia (Pan et al., 2014). Because there is a correlation between the expression levels of HOX proteins and the sensitivity to BCL2 inhibitor in AML patient samples, the aberrant expression of HOXA9 is the potential mechanism for BCL2-dependence of MLL leukemia (Brumatti et al., 2013; Kontro et al., 2017). Oncogenic MYC expression often leads to apoptosis, which needs to be alleviated by additional genetic events for leukemic cell survival (McMahon, 2014). Indeed, co-expression of HOXA9, BCL2, or SOX4 promoted MYC-mediated leukemogenesis, while MYC alone was insufficient to induce leukemia in vivo. Although the downstream mechanisms could not be addressed, SOX4 also exhibited anti-apoptotic effects on MYC-expressing cells. BCL2 was highly expressed in MLL fusion-mediated leukemia (Figure 5—figure supplement 2A,B), and many human MLL fusion leukemia cell lines were sensitive to a BCL-2 inhibitor (i.e. ABT-199)(Pan et al., 2014). High expression of SOX4 is correlated to poor survival in AML patients (Lu et al., 2017). These results support the key roles of BCL2 and SOX4 in the development of MLL leukemia. Ectopic expression of MYC and BCL2 induced lymphoid leukemia, whereas the MYC/SOX4 combination exclusively induced myeloid leukemia (Figure 6—figure supplement 1B), suggesting that BCL2 confers relatively stronger survival advantages in the lymphoid lineage than in the myeloid lineage in vivo, while SOX4 does in the myeloid lineage. Importantly, there was a partial effect of single-gene knockout of *Bcl2* and *Sox4* on leukemia initiation and maintenance. This indicates that MLL fusion proteins exploit multiple anti-apoptotic pathways and that blocking a single anti-apoptotic pathway may be insufficient to completely abrogate leukemic potential. Thus, simultaneously blocking multiple anti-apoptotic pathways may be required for efficient molecularly targeted therapy of HOXA9-expressing leukemia.

Our results also provide an insight into the mode of function of HOXA9. *HOX* genes are known to be expressed in a position-specific manner, conferring a positional identity to a cell (Wang et al., 2009). Our results suggest that HOXA9 unlikely functions as a major upstream factor determining tissue-specific gene expression by turning a silenced chromatin into transcriptionally active chromatin. Rather, HOX proteins likely play a supportive role in maintaining gene expression, which was activated by other transcriptional regulators. Our observation that HOXA9 cannot reactivate gene expression once silenced fits this hypothesis. Accordingly, HOXA9 maintains a subset of genes related to hematopoietic identity when expressed in hematopoietic precursors (Figure 2B). Recently, it has been reported that HOXA9 may recruit enhancer apparatuses, as it colocalizes with active enhancer mark (i.e. acetylated histone H3 lysine 27) (Sun et al., 2018). We speculate that HOXA9 may support the maintenance of an active enhancer, but unlikely establishes it on silenced chromatin. Further functional analysis of HOXA9 is required to understand how HOXA9 regulates gene expression.

In summary, our results describe the oncogenic roles for HOXA9 as a transcriptional maintenance factor for multiple anti-apoptotic genes, which are necessary to promote MYC-mediated leukemogenesis. In the case of MLL fusion-mediated leukemia, MLL fusion proteins directly activate both *MYC* and *HOXA9*, while HOXA9 maintains expression of *MYC*, *BCL2*, and *SOX4*, achieving high MYC activity and anti-apoptotic properties simultaneously (Figure 7E). Thus, MLL fusion-mediated LCs possess highly proliferative potentials and survival advantages using HOXA9 as a critical mediator.

## Materials and methods

### Vector constructs

For protein expression vectors, cDNAs obtained from Kazusa Genome Technologies Inc (Nagase et al., 2008) were modified by PCR-mediated mutagenesis and cloned into the pMSCV vector (for virus production) or pCMV5 vector (for transient expression) by restriction enzyme digestion and DNA ligation. The MSCV-neo MLL-ENL and MLL-AF10 vectors have been previously described (Okuda et al., 2017). sgRNA-expression vectors were constructed using the pLKO5.sgRNA.EFS.GFP vector (RRID:Addgene_57822) (Heckl et al., 2014). shRNA-expression vectors were purchased from Dharmacon. The target sequences are listed in Supplementary file 1.

### Cell lines

HEK293T cells were a gift from Michael Cleary and were authenticated by the JCRB Cell Bank in 2019 (Key Resources Table). HEK293TN cells were purchased from System Biosciences (RRID:CVCL_UL49). The cells were cultured in Dulbecco’s modified Eagle’s medium (DMEM) supplemented with 10% fetal bovine serum (FBS) and penicillin-streptomycin (PS). The Platinum-E (PLAT-E) ecotropic virus packaging cell line—a gift from Toshio Kitamura (RRID:CVCL_B488)(Morita et al., 2000)—was cultured in DMEM supplemented with 10% FBS, puromycin, blasticidin, and PS. The human leukemia cell lines including HB1119, (RRID:CVCL_8227), K562 (RRID:CVCL_0004), RS4-11 (RRID:CVCL_0093), THP1 (RRID:CVCL_0006), and EOL1 (RRID:CVCL_0258) were gifts from Michael Cleary (Tkachuk et al., 1992; Yokoyama et al., 2004), and were cultured in RPMI 1640 medium supplemented with 10% FBS and PS. The MV4-11 cell line (ATCC, RRID:CVCL_0064) was cultured in Iscove’s modified Dulbecco’s medium (IMDM) supplemented with 10% FBS and PS. The ML-2 cell line (DSMZ, RRID:CVCL_1418) was cultured in RPMI 1640 medium supplemented with 10% FBS and PS. MLL-ENL LCs (MLL-ENLbm0713) were described previously (Okuda et al., 2017). All the cell lines except HEK293TN, PLAT-E, and HB1119 were authenticated using short tandem repeat (STR)-PCR method by the JCRB Cell Bank. Cells were cultured in the incubator at 37°C and 5% CO_2_, and routinely tested for mycoplasma using the MycoAlert Mycoplasma detection kit (Lonza). 

### Animal models

For the in vivo leukemogenesis assay, 8-week-old female C57BL/6JJcl (C57BL/6J) or C. B-17/Icr-*scid/scid*Jcl (SCID) mice were purchased from CLEA Japan (Tokyo, Japan). *Bcl2*-knockout mice were a gift from Yoshihide Tsujimoto and provided via RIKEN BRC (Kamada et al., 1995). *Sox4*-knockout mice were a gift from Hans Clevers and provided via RIKEN BRC (Schilham et al., 1996).

### Western blotting

Western blotting was performed as previously described (Yokoyama et al., 2002). Antibodies used in this study are listed in Key Resources Table.

### Virus production

Ecotropic retrovirus constructed in pMSCV vectors was produced using PLAT-E packaging cells (Morita et al., 2000). Lentiviruses were produced in HEK293TN cells using the pMDLg/pRRE (RRID:Addgene_12251), pRSV-rev (RRID:Addgene_12253), and pMD2.G (RRID:Addgene_12259) vectors, all of which were gifts from Didier Trono (Dull et al., 1998). The virus-containing medium was harvested 24–48 hr following transfection and used for viral transduction.

### Myeloid progenitor transformation assay

The myeloid progenitor transformation assay was conducted as previously described (Lavau et al., 1997; Okuda and Yokoyama, 2017a). Bone marrow cells were harvested from the femurs and tibiae of 5-week-old female C57BL/6J mice. c-Kit^+^ cells were enriched using magnetic beads conjugated with an anti-c-Kit antibody (Miltenyi Biotec, RRID:AB_2753213), transduced with a recombinant retrovirus by spinoculation, and then plated (4 × 10^4^ cells/ sample) in a methylcellulose medium (IMDM, 20% FBS, 1.6% methylcellulose, and 100 µM β-mercaptoethanol) containing murine stem cell factor (mSCF), interleukin 3 (mIL-3), and granulocyte-macrophage colony-stimulating factor (mGM-CSF; 10 ng/mL each). During the first culture passage, G418 (1 mg/mL) or puromycin (1 μg/mL) was added to the culture medium to select for transduced cells. *Hoxa9* expression was quantified by qRT-PCR after the first passage. Cells were then re-plated once every 4–6 days with fresh medium; the number of plated cells for the second, third, and fourth passages was 4 × 10^4^, 2 × 10^4^, and 1 × 10^4^ cells/well, respectively. CFUs were quantified per 10^4^ plated cells at each passage.

### In vivo leukemogenesis assay

In vivo leukemogenesis assays were conducted as previously described (Lavau et al., 1997; Okuda and Yokoyama, 2017b). c-Kit^+^ cells (2 × 10^5^) prepared from the femurs and tibiae of 5-week-old female C57BL/6J mouse were transduced with retrovirus by spinoculation and intravenously transplanted into sublethally irradiated (5–6 Gy) C57BL/6J mice. For secondary leukemia, LCs (2 × 10^5^) cultured ex vivo for more than three passages were transplanted. As for knockouts of *Bcl2* and *Sox4*, mice heterozygous for *Bcl2* or *Sox4* were crossed, and c-Kit^+^ cells were isolated from fetal livers at E14–15 (for *Bcl2*) or E13 (for *Sox4*). The next day, cells were transduced with the retroviruses for MLL-AF10 or HOXA9/MEIS1 and transplanted intravenously into sublethally irradiated (2.5 Gy) SCID mice [2 × 10^5^ (for *Bcl2*) or 1 × 10^5^ cells/mouse (for *Sox4*)].

### qRT-PCR

Total RNA was isolated using the RNeasy Mini Kit (Qiagen) and reverse-transcribed using the Superscript III First Strand cDNA Synthesis System (Thermo Fisher Scientific) with oligo (dT) primers. Gene expression was analyzed by qPCR using TaqMan probes (Thermo Fisher Scientific). Relative expression levels were normalized to those of *GAPDH*/*Gapdh, ACTB/Actb,* or *TBP*/*Tbp* and determined using a standard curve and the relative quantification method, according to manufacturer’s instructions (Thermo Fisher Scientific). Commercially available/custom made PCR probes used are listed in Supplementary file 1.

### ChIP-qPCR and ChIP-seq

ChIP was performed as previously described (Okuda et al., 2017), using the fanChIP method (Miyamoto and Yokoyama, 2021). DNA was precipitated with glycogen, dissolved in TE buffer, and analyzed by qPCR (ChIP-qPCR) or deep sequencing (ChIP-seq). The qPCR probe/primer sequences are listed in Supplementary file 1. Deep sequencing was performed using the TruSeq ChIP Sample Prep Kit (Illumina) and HiSeq2500 (Illumina) at the core facility of Hiroshima University and described in our previous publication (Okuda et al., 2017).

### RNA-seq

Total RNA was prepared using the RNeasy Kit (Qiagen) and analyzed using a Bioanalyzer (Agilent Technologies). Deep sequencing was performed using a SureSelect Strand Specific RNA Library Prep Kit (Agilent Technologies) and GAIIx (Illumina) with 36 bp single-end reads or HiSeq2500 (Illumina) with 51 bp single-end reads at the core facility of Hiroshima University. Sequenced reads were mapped to the human genome assembly hg19 or the mouse genome assembly mm9 using CASAVA 1.8.2 (Illumina, RRID:SCR_001802), and read counts were normalized as reads per kilo base of exon per million mapped (RPKM). To define HOXA9 high and MYC high signature genes, data were trimmed by removing lowly expressed genes whose RPKM values were less than 2, and the relative expression between HOXA9-ICs and MYC-ICs was estimated. The top 50 HOXA9 high and MYC high signature genes were visualized as a heatmap using the Complex Heatmap package (RRID:SCR_017270)(Gu et al., 2016). To define HOXA9-MYC common target genes, lowly expressed genes were removed, and genes with more than two-fold expression compared to c-Kit^+^ cells were determined for each of HOXA9-ICs and MYC-ICs. The overlapping genes were defined as ‘HOXA9-MYC common target genes’. GSEA analysis and Kyoto Encyclopedia of Genes and Genomes (KEGG) pathway analysis were performed using the DAVID (RRID:SCR_001881)(Jiao et al., 2012), GSEA (Subramanian et al., 2005), and Metascape (Zhou et al., 2019).

### sgRNA competition assay

*Cas9* was introduced via lentiviral transduction using the pKLV2-EF1a-Cas9Bsd-W vector (RRID:Addgene_68343) (Tzelepis et al., 2016). Cas9-expressing stable lines were established with blasticidin (10–30 µg/mL) selection. The targeting sgRNA was co-expressed with GFP via lentiviral transduction using pLKO5.sgRNA.EFS.GFP vector (RRID:Addgene_57822)(Heckl et al., 2014). Percentages of GFP^+^ cells were initially determined by FACS analysis at 2 or 3 days after sgRNA transduction and then measured once every 3–5 days.

### FACS analysis and sorting

To detect apoptosis, two to five million cells were suspended in 200 µL of reaction buffer (140 mM NaCl, 10 mM HEPES, 2.5 mM CaCl_2_, and 0.1% BSA) and incubated with APC-Annexin V (RRID:AB_2868885) for 15 min and Propidium iodide for 5 min at room temperature. The cells were then centrifuged, resuspended in fresh reaction buffer, and analyzed with FACS Melody (BD Bioscience). FACS sorting of mouse bone marrow cells was performed with fluorophore-conjugated antibodies listed in Key Resources Table as previously described (Yokoyama et al., 2013).

### Accession numbers

Deep sequencing data used in this study have been deposited in the DNA Data Bank of Japan (DDBJ) Sequence Read Archive under the accession numbers listed in Supplementary file 1.

### Statistics

Statistical analyses were performed using GraphPad Prism seven software (RRID:SCR_002798). Data are presented as the mean with standard deviation (SD). Comparisons between two groups were analyzed by unpaired two-tailed Student’s *t*-test, while multiple comparisons were performed by ordinary one-way analysis of variance (ANOVA) followed by Dunnett’s test or two-way ANOVA. Mice transplantation experiments were analyzed by the log-rank test and Bonferroni correction was applied for multiple comparisons. p Values < 0.05 were considered statistically significant. n.s.: p>0.05, *: p ≤ 0.05, **: p ≤ 0.01, ***: p ≤ 0.001, and ****: p ≤ 0.0001.

### Study approval

All animal experimental protocols were approved by the National Cancer Center (Tokyo Japan) Institutional Animal Care and Use Committee.

## Data Availability

ChIP-seq data have been deposited to the DDBJ archive and have been published (accession number: DRA004871). RNA-seq data have been deposited to the DDBJ archive and have been published (accession number: DRA010090, DRA012078, DRA004874, DRA012079). The following datasets were generated: HIROSHIMA2020Expression profiles of murine myeloid progenitors immortalized by various oncogenesDDBJ GEAE-GEAD-360 HIROSHIMA2021Expression profiles of murine myeloid progenitors immortalized by various oncogenesDDBJ GEAE-GEAD-435 HIROSHIMA2021Expression profiles of human cell linesDDBJ GEAE-GEAD-436 HIROSHIMA2018Sequence reads of murine myeloid progenitors immortalized by various oncogenesDDBJ DRADRA010090 HIROSHIMA2015Sequence reads of murine myeloid progenitors immortalized by various oncogenesDDBJ DRADRA012078 HIROSHIMA2016Sequence reads of various factors/modificaions in HB1119 cellsDDBJ DRADRA004874 HIROSHIMA2015Sequence reads of human cell linesDDBJ DRADRA012079 HIROSHIMA2020Expression profiles of HB1119 and 293T cell linesDDBJ GEAE-GEAD-321 The following previously published datasets were used: HIROSHIMA2016Genomic localization of various factors/modificaions in HB1119 cellsDDBJDRA004871 HIROSHIMA2020Genomic localization of various factors/modificaions in HB1119 cellsDDBJ GEAE-GEAD-319

## Additional files

Source data 1. Gel images.

Supplementary file 1. Reagents and data sets.

Transparent reporting form

## Appendix 1

**Appendix 1—key resources table:**

Reagent type (species) or resource	Designation	Source or reference	Identifiers	Additional information
Strain, strain background (*Mus musculus*)	C57BL/6J	CLEA Japan		
Strain, strain background (*Mus musculus*)	Bcl2 KO (B6.129P2-Bcl2<tm1Tsu>/TsuRbrc)	Gift from Yoshihide Tsujimoto (via RIKEN BRC) Kamada et al., 1995		
Strain, strain background (*Mus musculus*)	Sox4 KO (STOCK Sox4<tm1Cle>/Mmmh)	Gift from Hans Clevers (via RIKEN BRC) Schilham et al., 1996		
Cell line (Homo-sapiens)	PLAT-E	Gift from Toshio Kitamura Morita et al., 2000	RRID:CVCL_B488	
Cell line (Homo-sapiens)	HB1119	Gift from Michael L. Cleary Tkachuk et al., 1992; Yokoyama et al., 2004		The cell line was verified by the expression of MLL-ENL
Cell line (Homo-sapiens)	HEK293TN	System Bioscience	Cat# LV900A-1 RRID:CVCL_UL49	
Cell line (Homo-sapiens)	HEK293T	Gift from Michael L. Cleary Yokoyama et al., 2004		authenticated by the JCRB Cell Bank in 2019
Cell line (Homo-sapiens)	K562	Gift from Michael L. Cleary Yokoyama et al., 2004	RRID:CVCL_0004	authenticated by the JCRB Cell Bank in 2021
Cell line (Homo-sapiens)	MV4-11	ATCC	Cat# CRL-9591 RRID:CVCL_0064	authenticated by the JCRB Cell Bank in 2021
Cell line (Homo-sapiens)	RS4-11	Gift from Michael L. Cleary	RRID:CVCL_0093	authenticated by the JCRB Cell Bank in 2021
Cell line (Homo-sapiens)	ML-2	DSMZ	Cat# ACC15 RRID:CVCL_1418	authenticated by the JCRB Cell Bank in 2021
Cell line (Homo-sapiens)	THP1	Gift from Michael L. Cleary	RRID:CVCL_0006	authenticated by the JCRB Cell Bank in 2021
Cell line (Homo-sapiens)	EOL-1	Gift from Michael L. Cleary	RRID:CVCL_0258	authenticated by the JCRB Cell Bank in 2021
Antibody	MLLn ab#1 (Rabbit polyclonal)	In-house Yokoyama et al., 2002	rpN1	ChIP (1:400)
Antibody	MLLn ab#2 (Rabbit monoclonal, D2M7U)	Cell Signaling Technology	Cat# 14689 RRID:AB_2688009	ChIP (1:400)
Antibody	MLLc (Rabbit monoclonal, D6G8N)	Cell Signaling Technology	Cat# 14197 RRID:AB_2688010	WB (1:1000)
Antibody	MENIN (Rabbit polyclonal)	Bethyl Laboratories	Cat# A300-105A RRID:AB_2143306	ChIP (1:400) WB (1:1000)
Antibody	MYC (Rabbit monoclonal, D3N8F)	Cell Signaling Technology	Cat# 13987 RRID:AB_2631168	WB (1:2000)
Antibody	Caspase3 (Rabbit monoclonal, D3R6Y)	Cell Signaling Technology	Cat# 14220 RRID:AB_2798429	WB (1:2000)
Antibody	Cleaved Caspase3 (Rabbit monoclonal, 5A1E)	Cell Signaling Technology	Cat# 9664 RRID:AB_2070042	WB (1:2000)
Antibody	PARP (Rabbit polyclonal)	Cell Signaling Technology	Cat# 9542 RRID:AB_2160739	WB (1:2000)
Antibody	γH2AX (Rabbit polyclonal)	Bethyl Laboratories	Cat# A300-081A RRID:AB_203288	WB (1:1000)
Antibody	BCL2 (Mouse monoclonal, C-2)	Santa Cruz Biotechnology	Cat# sc-7382 RRID:AB_626736	WB (1:500)
Antibody	SOX4 (Mouse monoclonal, B-7)	Santa Cruz Biotechnology	Cat# sc-518016	WB (1:200)
Antibody	HOXA9 (Rabbit polyclonal)	Millipore	Cat# 07–178 RRID:AB_11210179	WB (1:1000)
Antibody	GAPDH (Rabbit polyclonal)	Santa Cruz Biotechnology	Cat# sc-25778 RRID:AB_10167668	WB (1:2000)
Antibody	BAK (Rabbit monoclonal, D4E4)	Cell Signaling Technology	Cat# 12105 RRID:AB_2716685	WB (1:2000)
Antibody	BAD (Rabbit monoclonal, D24A9)	Cell Signaling Technology	Cat# 9239 RRID:AB_2062127	WB (1:2000)
Antibody	BCL-XL (Rabbit monoclonal, 54H6)	Cell Signaling Technology	Cat# 2764 RRID:AB_2228008	WB (1:2000)
Antibody	MCL1 (Rabbit monoclonal, D35A5)	Cell Signaling Technology	Cat# 5453 RRID:AB_10694494	WB (1:2000)
Antibody	APC Annexin V	BD Biosciences	Cat# 550475 RRID:AB_2868885	FACS (1:100)
Antibody	Propidium iodide	Thermo Fisher Scientific	P3566	FACS (1:2000)
Antibody	Gr-1 (Rat monoclonal, RB6-8C5)	BD Biosciences	Cat# 553127 RRID:AB_394643	FACS (1:100)
Antibody	B220 (Rat monoclonal, RA3-6B2)	BD Biosciences	Cat#: 553088 RRID:AB_394618	FACS (1:100)
Antibody	TER119 (Rat monoclonal, TER-119)	BD Biosciences	Cat#: 557915 RRID:AB_396936	FACS (1:100)
Antibody	CD3e (Hamster monoclonal, 145–2 C11)	BD Biosciences	Cat#: 553062 RRID:AB_394595	FACS (1:100)
Antibody	Mac/CD11b (Rat monoclonal, M1/70)	BD Biosciences	Cat#: 553310 RRID:AB_394774	FACS (1:100)
Antibody	c-Kit (Rat monoclonal, 2B8)	BD Biosciences	Cat#: 553355 RRID:AB_394806	FACS (1:100)
Antibody	Sca1 (Rat monoclonal, D7)	BD Biosciences	Cat#: 553108 RRID:AB_394629	FACS (1:100)
Antibody	CD34 (Rat monoclonal, RAM34)	BD Biosciences	Cat#: 751621 RRID:AB_2875614	FACS (1:100)
Antibody	CD16/32 (Rat monoclonal, 93)	BD Biosciences	Cat#: 751690 RRID:AB_2875675	FACS (1:100)
Recombinant DNA reagent	pMSCV-neo	Clontech	Cat#: 634401	Gene expression vector
Recombinant DNA reagent	pMSCV-puro	Clontech	Cat#: 634401	Gene expression vector
Recombinant DNA reagent	pMSCV-hygro	Clontech	Cat#: 634401	Gene expression vector
Recombinant DNA reagent	pLKO5.EFS.GFP	Addgene (gift from Benjamin Ebert) Heckl et al., 2014	Addgene Plasmid #57822 RRID:Addgene_57822	sgRNA backbone
Recombinant DNA reagent	pKLV2-Cas9.bsd	Addgene (gift from Kosuke Yusa) Tzelepis et al., 2016	Addgene Plasmid #68343 RRID:Addgene_68343	Cas9 expression vector
Recombinant DNA reagent	pMDLg/pRRE	Addgene (gift from Didier Trono) Dull et al., 1998	Addgene Plasmid #12251 RRID:Addgene_12251	Lentivirus packaging vector
Recombinant DNA reagent	pRSV-rev	Addgene (gift from Didier Trono) Dull et al., 1998	Addgene Plasmid #12253 RRID:Addgene_12253	Lentivirus packaging vector
Recombinant DNA reagent	pMD2.G	Addgene (gift from Didier Trono) Dull et al., 1998	Addgene Plasmid #12259 RRID:Addgene_12259	Lentivirus packaging vector
Recombinant DNA reagent	pLKO.1-puro	Addgene (gift from Bob Weinberg) Stewart et al., 2003	Addgene Plasmid #84530 RRID:Addgene_84530	shRNA backbone
Sequence-based reagent	sgRNAs	This study		See Supplementary file 1
Sequence-based reagent	shRNA	This study		See Supplementary file 1
Sequence-based reagent	qPCR primers	Thermo Fisher Scientific		See Supplementary file 1
Commercial assay or kit	RNeasy Mini Kit	Qiagen	Cat#: 74106	
Commercial assay or kit	Super Script III First-Strand Synthesis System	Thermo Fisher Scientific	Cat# 18080051	
Commercial assay or kit	TruSeq ChIP Sample Prep Kit SetB	Illumina	Cat# IP-202–1024	
Commercial assay or kit	Sure Select Strand Specific RNA Library Prep Kit	Agilent Technologies	Cat#: G9691A	
Software, algorithm	GraphPad Prism7	GraphPad Software Inc	RRID:SCR_002798	Data analysis
Software, algorithm	FlowJo	BD Biosciences	RRID:SCR_008520	FACS data analysis
Software, algorithm	Integrative Genomics Viewer	Thorvaldsdóttir et al., 2013	RRID:SCR_011793	Data visualization
Software, algorithm	CASAVA 1.8.2	Illumina	RRID:SCR_001802	RNA-seq data analysis
Software, algorithm	DAVID	Jiao et al., 2012	RRID:SCR_001881	RNA-seq data analysis
Software, algorithm	Complex heatmap	Gu et al., 2016	RRID:SCR_017270	RNA-seq data analysis
Software, algorithm	R2: Genome Analysis and Visualization Platform	AMC: Oncogenomics	http://r2.amc.nl http://r2platform.com	RNA-seq data analysis
Software, algorithm	Gene set enrichment analysis	Subramanian et al., 2005	https://www.gsea-msigdb.org/gsea/index.jsp	RNA-seq data analysis
Other	c-Kit magnetic beads	Miltenyi Biotec	Cat# 130-091-224 RRID:AB_2753213	
