# Supplementary material for: HOXA9 promotes MYC-mediated leukemogenesis by maintaining gene expression for multiple anti-apoptotic pathways
Source: eLife. 2021 Jul 26;10:e64148. doi: 10.7554/eLife.64148 (PMC8313233; doi:10.7554/eLife.64148)

Figure 3

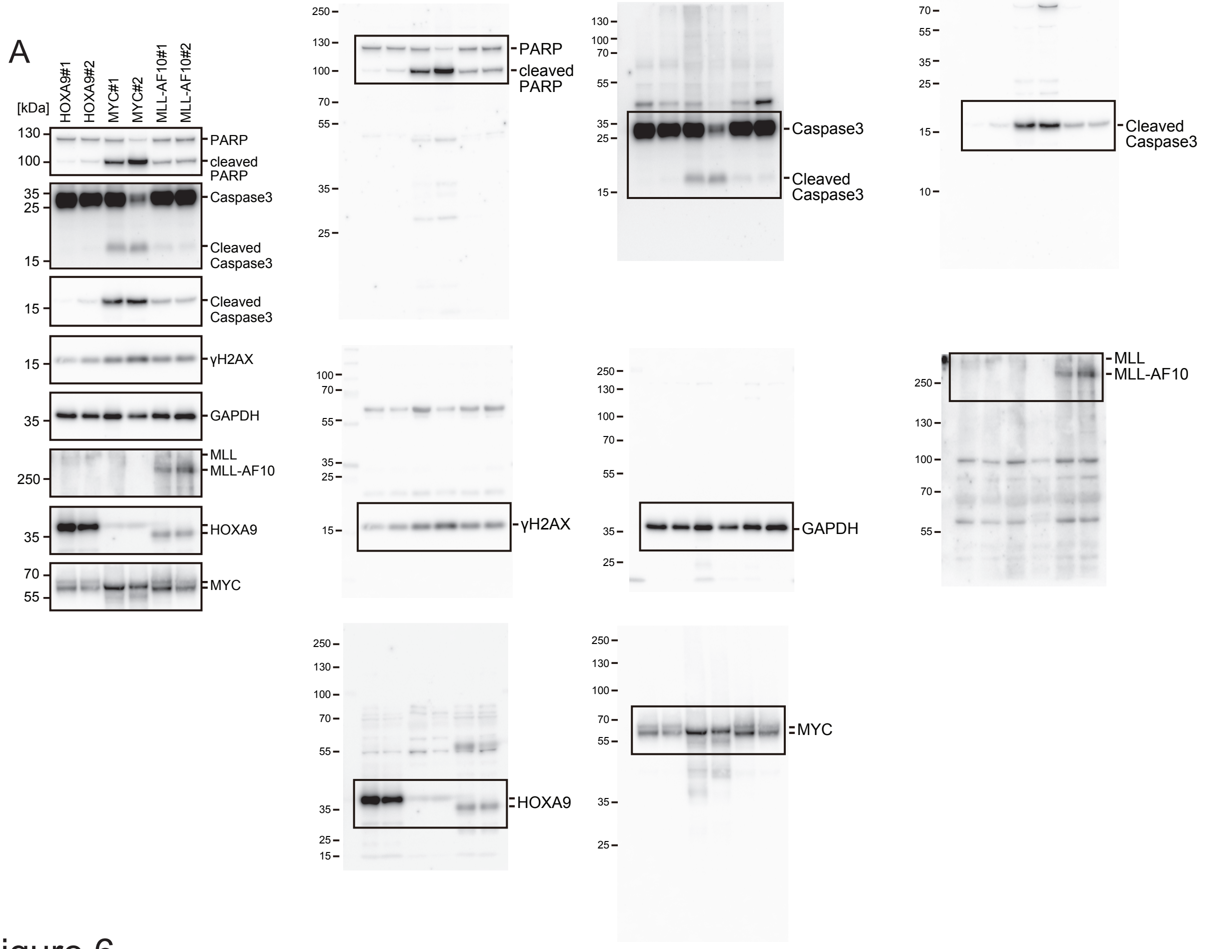

Figure 6

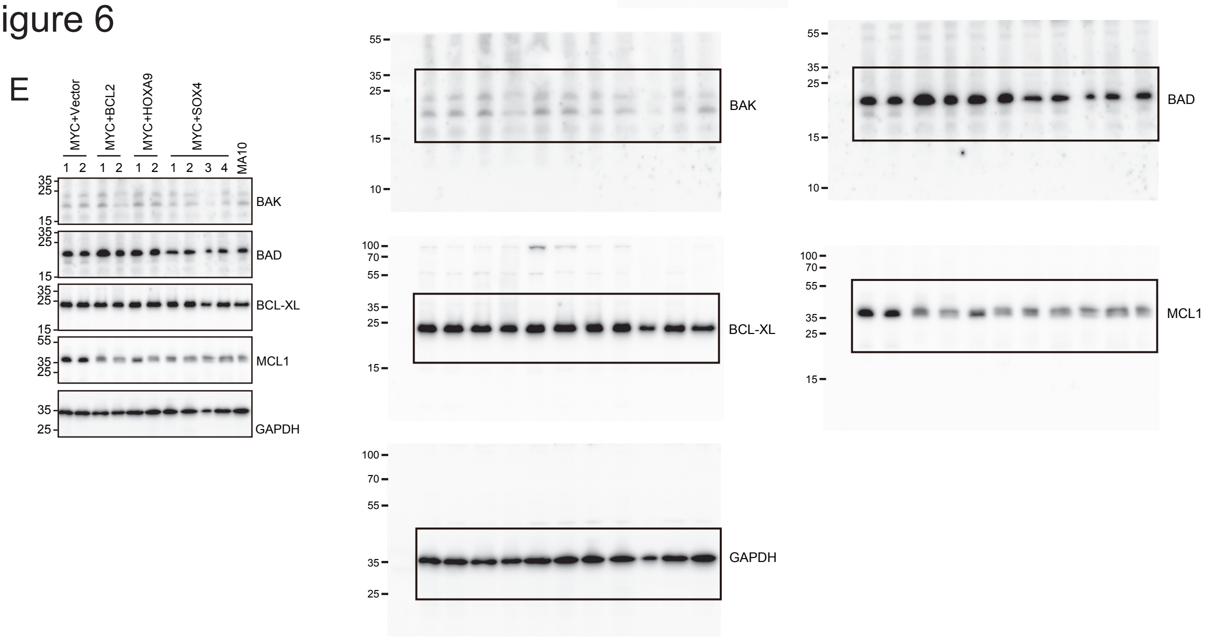

Figure 7

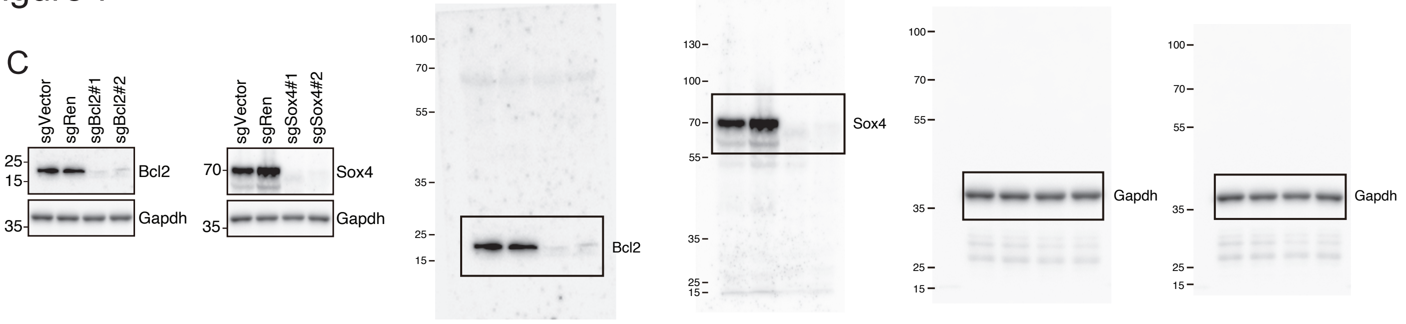

Figure 1-figure supplement 1

B

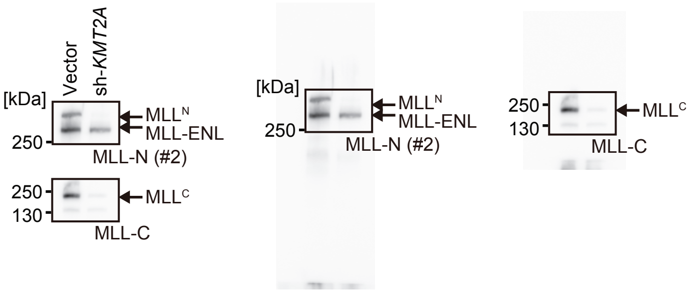

Figure 1-figure supplement 2

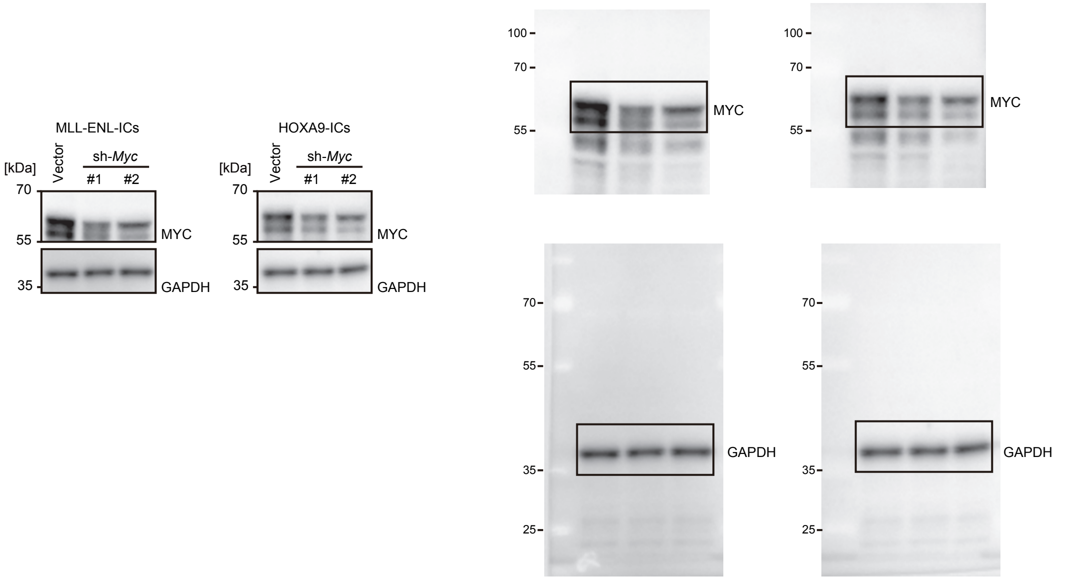

Figure 6-figure supplement 1

A

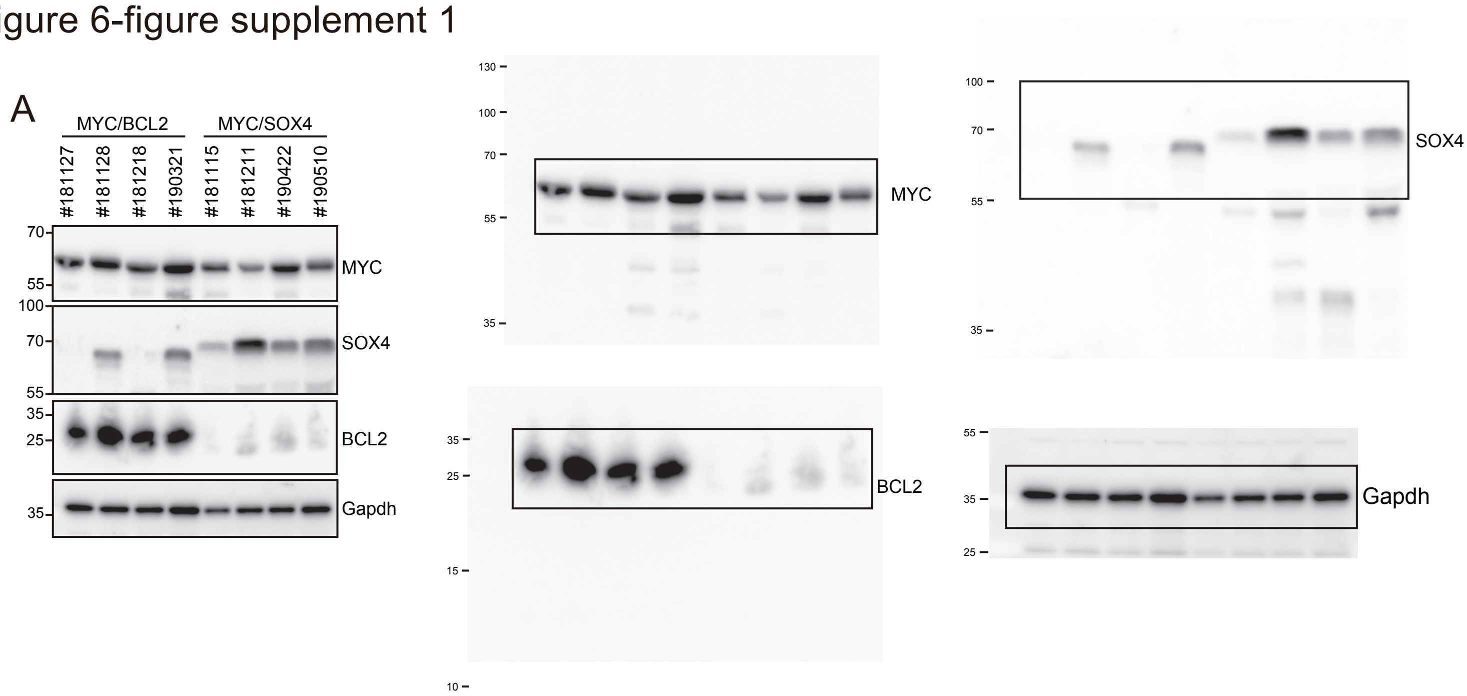

Figure 7-figure supplement 2

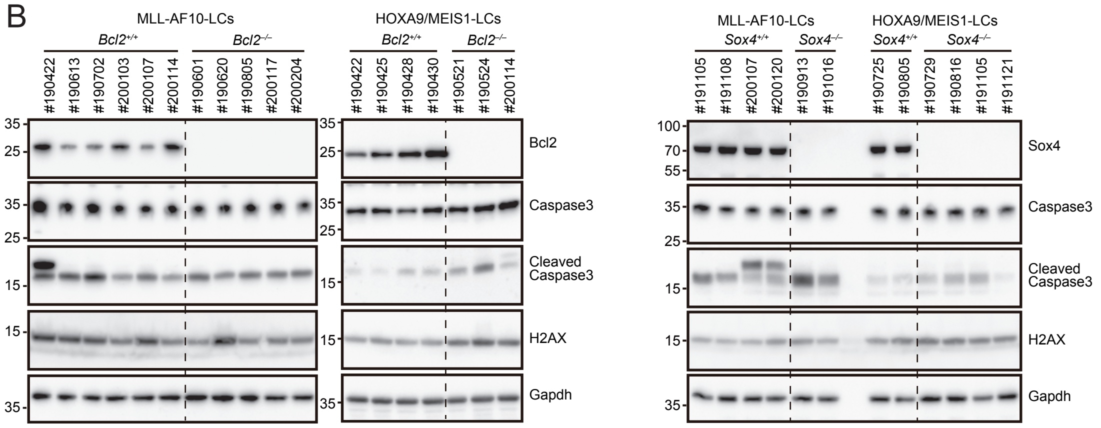

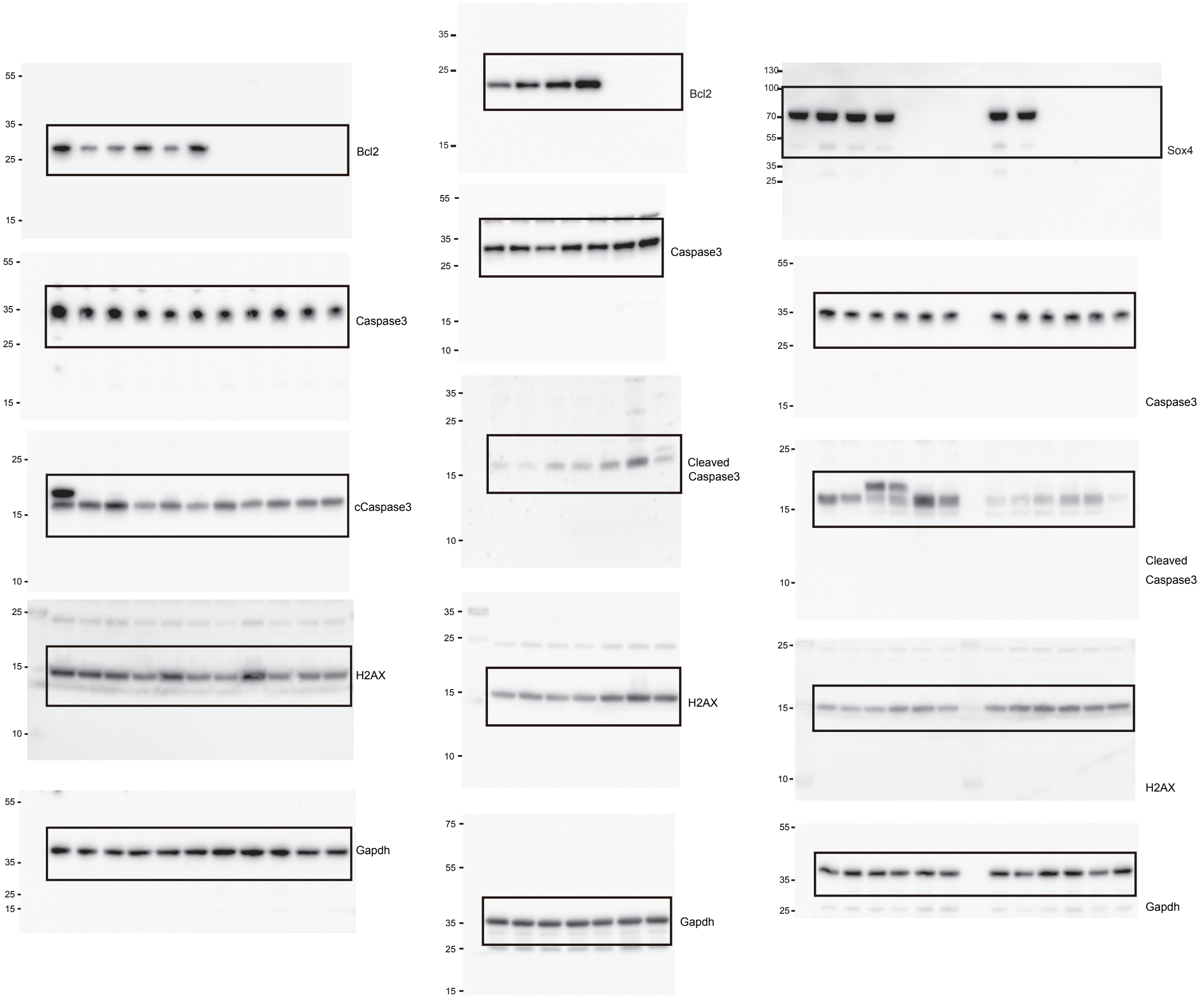

Supplement: Source data 1. [file elife-64148-data1.pdf]
