# Supplementary material for: HOXA9 promotes MYC-mediated leukemogenesis by maintaining gene expression for multiple anti-apoptotic pathways
Source: eLife. 2021 Jul 26;10:e64148. doi: 10.7554/eLife.64148 (PMC8313233; doi:10.7554/eLife.64148)
Supplement: Supplementary file 1. [file elife-64148-supp1.docx]

Supplementary file 1

sgRNA target sequences

| Target name | Target sequence |
| --- | --- |
| Bcl2#1 | GCCCGCTGTGCACCGGGACA |
| Bcl2#2 | GGTCCATCTGACCCTCCGCC |
| Sox4#1 | CGCGTCGACGGGCGGCAAGG |
| Sox4#2 | GATCGAGCGGCGCAAGATCA |
| Men1 | TCTGCGCTCTATCGACGACG |
| Ren | GGATGATAACTGGTCCGCAG |

shRNA target sequences

| Target name | Target sequence | Identifier |
| --- | --- | --- |
| Myc#1 | CGAGAACAGTTGAAACACAAA | TRCN0000042513 |
| Myc#2 | CGACGAGGAAGAGAATTTCTA | TRCN0000042515 |
| MLL | TGCCAAGCACTGTCGAAATTA | TRCN0000234743 |

qPCR primers

| Target name | Forward | Reverse | Reporter |
| --- | --- | --- | --- |
| PITX2 pre-TSS | AGGGCAGTTGCTCTGAAGTC | CTGCAGAAGGAGCTCTTGGA | ACTGCCTGGCCACTCC |
| PITX2 TSS | AGAGAGAGTGCGAGACCGA | GCCACTGGCAGTTTCTTTCTG | CCTCTCCAGCTTTCTC |
| PITX2 post-TSS | CAGGCCCAAGCGAATTACCT | AGTTGACTGGTGATCAATTTAAAGGAGTT | CTGGATGCCAAGCTCT |
| HOXA7 pre-TSS | GCCTTCCCCGTCTGGAT | ACTCTGCCCAAGTCTTCTCTCA | CAGGCCGGACTTAGAC |
| HOXA7 TSS | GACGCCTACGGCAACCT | GCCTTTGGCGAGGTCACT | CCCTGCGCCTCCTAC |
| HOXA7 post-TSS | TGCCAGGGTCCATTTCAAGATG | CCCTCATCCCCAGGACCTT | CTCTGTCCTCATTCCC |
| HOXA9 pre-TSS | TGGCTGCTTTTTTATGGCTTCAATT | CCGCGTGCGAGTGC | CCCCTCACATAAAATT |
| HOXA9 TSS | TCACCACCACCCCTACGT | GCAAGCCCGCGAAGGA | CAGGAGCGCATGTACC |
| HOXA9 post-TSS | AGTGGCGGCGTAAATCCT | TGATCACGTCTGTGGCTTATTTGAA | CCCGCAGCCTCATC |
| HOXA10 pre-TSS | GGGCCGTCTTTCCATCAAG | AACTCCTGTCAGTGGACTTTGG | CAGTCCCGCCAGCCCA |
| HOXA10 TSS | GGCGGTGGCGGTTACTAC | CGACGCTGCCTCATTGC | CTGCCCTACGGGCTGC |
| HOXA10 post-TSS | CCTCAGCTCCCCCTGACTAG | CCCGGCCACAGGAAAGAG | CAGCGAGCAGGCCCCCT |
| MEIS1 pre-TSS | CGGCGTTGATTCCCAATTTATTTCA | CACACAAACGCAGGCAGTAG | CCGCCAGCTTTATTTT |
| MEIS1 TSS | TTTGCTTCAGGTCCCGTAGAC | CCTTAACGTCTCCAGCAACGT | ACTGGTCCCAGATCTT |
| MEIS1 post-TSS | TCTCAGCGCCTCCAAATCTTG | TTTGTGTGTGTGAAATTTAGCTATTTAGGTTTT | CCAGGCAGTTATTTTC |
| MYC pre-TSS | GCGGTATCTGCTGCTTTGG | GCATTATGTATGCACAGCTATCTGGAT | CTGGGTGGAAGGTATCC |
| MYC TSS | CCGGCTAGGGTGGAAGAG | GAGGCGAAGCCCCCTATTC | CAGGACGCCCGCAGCG |
| MYC post-TSS | GGGTAGGCGCAGGCA | GGTTTTTCCAAGTCAACGATTCCA | ATGTGTCCGATTCTCC |

Probes for RT-qPCR (mouse)

| Target name (Mouse) | Source | Identifier |
| --- | --- | --- |
| Bcl2 | Thermo Fisher Scientific | Mm00477631_m1 |
| Bcl2a1b | Thermo Fisher Scientific | Mm03646861_mH |
| Bcl2l1 | Thermo Fisher Scientific | Mm00437783_m1 |
| Bcl2l2 | Thermo Fisher Scientific | Mm00432054_m1 |
| Gapdh | Thermo Fisher Scientific | Mm99999915_g1 |
| Hoxa10 | Thermo Fisher Scientific | Mm00433966_m1 |
| Hoxa9 (Hoxa9/HOXA9) | Thermo Fisher Scientific | Mm00439364_m1 |
| Igf1 | Thermo Fisher Scientific | Mm00439560_m1 |
| Itgam (Cd11b) | Thermo Fisher Scientific | Mm00434455_m1 |
| Max | Thermo Fisher Scientific | Mm00484802_g1 |
| Mcl1 | Thermo Fisher Scientific | Mm01257351_g1 |
| Meis1 (Meis1/MEIS1) | Thermo Fisher Scientific | Mm00487664_m1 |
| Mxd1 | Thermo Fisher Scientific | Mm00487504_m1 |
| Myc | Thermo Fisher Scientific | Mm00487804_m1 |
| Sox4 | Thermo Fisher Scientific | Mm00486320_s1 |
| Tbp | Thermo Fisher Scientific | Mm01277042_m1 |

Probes for RT-qPCR (human)

| Target name (Human) | Source | Identifier |
| --- | --- | --- |
| HOXA9 | Thermo Fisher Scientific | Hs00365969_m1 |
| Meis1 (Meis1/MEIS1) | Thermo Fisher Scientific | Mm00487664_m1 |
| MYC | Thermo Fisher Scientific | Hs1570247_m1 |

Probe for RT-qPCR primer (custom, human)

| Target name | Source | Forward | Reverse | Reporter |
| --- | --- | --- | --- | --- |
| MLL | Thermo Fisher Scientific | CCAAGTTTGGTGGTCGCAATATAAA | CATCCATTGTAGATTCTGACATTTT | CAGTGCTGCAAGATGAG |

Accession numbers of ChIP-seq data

| Sample name | DRA accession number | Sample ID |
| --- | --- | --- |
| HB1119-fanChIP-INPUT | DRA004871 | SAMD00055689 |
| HB1119-fanChIP-MLLn ab#1 | DRA004871 | SAMD00055685 |
| HB1119-fanChIP-MLLn ab#2-pLKO1 | DRA004871 | SAMD00055687 |
| HB1119-fanChIP-MLLn ab#2-sh-MLL | DRA004871 | SAMD00055688 |

Accession numbes of mRNA-seq data

| Sample name | DRA accession number | Sample ID |
| --- | --- | --- |
| MP-fMLL-AF10-RNA 150916 | DRA010090 | SAMD00220959 |
| MP-fMLL-AF10-RNA 170719-1 | DRA010090 | SAMD00220960 |
| MP-fMLL-AF10-RNA 170719-2 | DRA010090 | SAMD00220961 |
| MP-fHOXA9-RNA 140824 | DRA010090 | SAMD00220962 |
| MP-fHOXA9-RNA 150812 | DRA010090 | SAMD00220963 |
| MP-fHOXA9-RNA 170719-1 | DRA010090 | SAMD00220964 |
| MP-fMYC-RNA 131204 | DRA010090 | SAMD00220965 |
| MP-fMYC-RNA 141113 | DRA010090 | SAMD00220966 |
| MP-fMYC-RNA 170719 | DRA010090 | SAMD00220967 |
| cKit-positive (day0)-RNA | DRA012078 | SAMD00324538 |
| LC-MYC/HOXA9 (BM0317)-RNA | DRA012078 | SAMD00324539 |
| LC-HOXA9/MEIS1 (BM0707A)-RNA | DRA012078 | SAMD00324540 |
| HEK293T-RNA | DRA004874 | SAMD00055715 |
| K562-RNA | DRA012079 | SAMD00324596 |
| HB1119-RNA#1 | DRA004874 | SAMD00055714 |
| HB1119-RNA#2 | DRA012079 | SAMD00324597 |
| MV4-11-RNA | DRA012079 | SAMD00324598 |
| RS4-11-RNA | DRA012079 | SAMD00324599 |
| ML-2-RNA | DRA012079 | SAMD00324600 |
| THP-1-RNA | DRA012079 | SAMD00324601 |
| EOL-1-RNA | DRA012079 | SAMD00324602 |
